# The effect of omega-3 fatty acids and its combination with statins on lipid profile in patients with hypertriglyceridemia: A systematic review and meta-analysis of randomized controlled trials

**DOI:** 10.3389/fnut.2022.1039056

**Published:** 2022-10-13

**Authors:** Yunjiao Yang, Wen Deng, Yanmei Wang, Tongyi Li, Yiding Chen, Cong Long, Qing Wen, Yue Wu, Qiu Chen

**Affiliations:** ^1^Hospital of Chengdu University of Traditional Chinese Medicine, Chengdu, China; ^2^School of Clinical Medicine, Chengdu University of Traditional Chinese Medicine, Chengdu, China; ^3^Mianyang Attached Hospital of Chengdu University of Traditional Chinese Medicine, Mianyang, China

**Keywords:** Omega-3 fatty acids, statins, triglyceride, low-density lipoprotein cholesterol, hypertriglyceridemia, meta-analysis

## Abstract

**Background/Aim:**

Omega-3 fatty acids (OM3-FA), a promising treatment for high triglycerides, have gradually attracted public attention. However, some studies showed that their application presented tricky problems, like increasing low-density lipoprotein cholesterol (LDL-C) levels. This study aimed to systematically evaluate the effect of OM3-FA or their combination with statins on the lipid profile in patients with hypertriglyceridemia.

**Materials and methods:**

This study followed the preferred reporting items for systematic reviews and meta-analyses (PRISMA 2020) guidelines. PubMed, Embase, Web of science, and Cochrane library were searched up to May 15, 2022. The random-effects model was applied to calculate the mean difference (MD) and associated 95% confidence intervals (CI).

**Results:**

This meta-analysis included 32 studies with 15,903 subjects. When OM3-FA was used as monotherapy compared with placebo, it significantly decreased TG (MD: −39.81, 95% CI: −54.94 to −24.69; *p* < 0.001), TC (MD: −2.98, 95% CI: −5.72 to −0.25, *p* = 0.03), very low-density lipoprotein cholesterol (VLDL-C) (MD: −25.12, 95% CI: −37.09 to −13.14; *p* < 0.001), and non-high-density lipoprotein cholesterol (non-HDL-C) levels (MD: −5.42, 95% CI: −8.06 to−2.78; *p* < 0.001), and greatly increased LDL-C (MD: 9.10, 95% CI: 4.27 to 13.94; *p* < 0.001) and HDL levels (MD: 1.60, 95% CI: 0.06 to 3.15; *p* = 0.04). Regarding apolipoprotein B (Apo-B) and apolipoprotein AI (Apo-AI), no significant effect was identified. When OM3-FA was combined with statins, significant reductions were observed in the concentrations of TG (MD: −29.63, 95% CI: −36.24 to −23.02; *p* < 0.001), TC (MD: −6.87, 95% CI: −9.30 to −4.45, *p* < 0.001), VLDL-C (−20.13, 95% CI: −24.76 to −15.50; *p* < 0.001), non-HDL-C (MD: −8.71, 95% CI: −11.45 to −5.98; *p* < 0.001), Apo-B (MD: −3.50, 95% CI: −5.37 to −1.64; *p* < 0.001), and Apo-AI (MD: −2.01, 95% CI: −3.07 to −0.95; *p* < 0.001). However, the combined therapy did not exert significant changes on the levels of high-density lipoprotein cholesterol (HDL-C) and LDL-C compared to control group.

**Conclusion:**

The use of OM3-FA either as monotherapy or in combination with statins may potentially reduce the levels of TG, TC, VLDL-C, non-HDL-C, Apo-B, and Apo-AI while increasing the levels of LDL-C and HDL-C. Nevertheless, the effects of OM3-FA observed in this review should be interpreted with caution due to the high heterogeneity between the included studies.

**Systematic review registration:**

[https://www.crd.york.ac.uk/prospero/], identifier [CRD42022329552].

## Introduction

Hypertriglyceridemia, one of the most prevalent diseases, is still a significant public health problem worldwide. A series of complications caused by hypertriglyceridemia are eroding people’s health. Studies showed that the incidence of cardiovascular events remains high in patients with cardiovascular disease even after primary or secondary preventive treatment. A growing body of literature demonstrated that hypertriglyceridemia as an independent risk factor for atherosclerotic cardiovascular disease (ASCVD) might explain this problem (1–6). In addition, hypertriglyceridemia is closely related to obesity, type 2 diabetes, nephrotic syndrome, chronic renal insufficiency, hypothyroidism, fatty liver, and ischemic stroke (7–9). Moreover, hypertriglyceridemia is an essential cause of pancreatitis. Pedersen et al. reported that mild-to-moderate hypertriglyceridemia from 177 mg/dl and above is associated with a high risk of acute pancreatitis (10). Therefore, it is so important to control triglycerides in a reasonable range. However, how to choose an appropriate treatment is still a problem to be solved.

In recent years, omega-3 fatty acids (OM3-FA) have gradually attracted public attention as a promising way to treat high triglycerides. A large proportion of scientific research has shown that OM3-FA could reduce triglyceride levels (11–14). Two meta-analyses evaluating the effects of OM3-FA on highly active antiretroviral therapy (HAART) associated hypertriglyceridemia in HIV/AIDS patients showed that OM3-FA significantly reduced triglyceride levels (15, 16). Moreover, OM3-FA has been approved in the United States to control hypertriglyceridemia (17). However, it was reported that the application of OM3-FA increased low-density lipoprotein cholesterol (LDL-C) (14, 18, 19), an essential risk factor for cardiovascular disease, and decreased apolipoprotein AI (Apo-AI) (18, 20, 21), a good lipoprotein. Therefore, a dilemma was posed regarding the use of OM3-FA. In addition, it is unclear whether such a problem exists in patients with hypertriglyceridemia treated with OM3-FA in combination with statins. A previous meta-analysis, including patients with hypertriglyceridemia (≥150 mg/dl), evaluated the effect of OM3-FA on triglycerides (22). Although this analysis demonstrated that OM3-FA could reduce triglycerides, the quality of literature was low, and the amount of sample size was not enough. Besides, the study was limited in assessing levels of lipids other than triglycerides. In recent years, many large randomized controlled, double-blind trials of OM3-FA for hypertriglyceridemia have been published (19, 20, 23, 24), providing further strong support for the determination of clinical evidence. In addition, no systematic review or meta-analysis has reported the effect of OM3-FA added to statins on serum lipid profile in patients with hypertriglyceridemia.

Based on the above existing problems, we conducted this systematic review and meta-analysis for the following purposes: first, to systematically evaluate the efficacy of OM3-FA, eicosapentaenoic acid (EPA), and docosahexaenoic acid (DHA) for patients with hypertriglyceridemia but not in AIDS-associated hypertriglyceridemia to provide reliable evidence for clinical practice; Second, to investigate whether OM3-FA affect other lipid levels in patients with hypertriglyceridemia; Third, to evaluate the effect of OM3-FA added to statins on lipid profile in patients with hypertriglyceridemia.

## Methods

This meta-analysis followed Preferred Reporting Items for Systematic Reviews and Meta-Analyses (PRISMA) guidelines (25). The PRISMA checklist is available in Supplementary material 1. We registered this meta-analysis on the PROSPERO database^1^ with the Registration Number (CRD42022329552).

### Search strategy

Two reviewers (YY and WD) independently searched databases including PubMed, Cochrane library, Embase, and Web of science from their inception to May 15, 2022. In addition, we also searched the Clinical Trials,^2^ unpublished gray literature, and references cited in the eligible studies. The key search terms are as follows: (“Omega-3 fatty acid” OR “n-3 Oil” OR “n-3 OM3-FA” OR “n-3 PUFA” OR “n-3 polyunsaturated fatty Acid” OR “eicosapentaenoic acid” OR “docosahexaenoic acids”) AND (“hypertriglyceridemia” OR “hyperlipidemia” OR “hyperlipemia”) AND (“randomized controlled trial” OR “RCT” OR “randomized clinical trial” OR “randomly”) Full details of the search strategy are available in Supplementary material 2.

### Inclusion criteria

*Population*: This study only included adults with triglyceride levels ≥150 mg/dl, without restriction on gender. A study was also eligible if the study participants had mixed hyperlipidemia, obesity, and metabolic syndrome as long as the triglyceride level was ≥150 mg/dl. *Intervention*: OM3-FA monotherapy or combined therapy of statins plus OM3-FA. The type of OM3-FA considered in this review included EPA and/or DHA. The OM3-FA should only be taken orally in the form of capsule with no minimum/maximum dose restriction. *Comparison*: For the OM3-FA monotherapy, the control group must be the placebo. Regarding the combined therapy of statins plus OM3-FA, the control group must also use statins with or without a placebo. There are no restrictions on the types of statins used. *Outcomes*: The primary efficacy parameter was triglyceride (TG) percentage change from baseline to end of the study; Secondary endpoints included total cholesterol (TC), high-density lipoprotein cholesterol (HDL-C), LDL-C, very low-density lipoprotein cholesterol (VLDL-C), non-high-density lipoprotein cholesterol (non-HDL-C), apolipoprotein B (Apo-B) and Apo-AI percentage change from baseline to end of the study. *Study design*: Randomized controlled trials with parallel design published in English. There was no restriction on the sample size and the intervention time.

### Exclusion criteria

*Population*: Patients with HIV/AIDS-associated hypertriglyceridemia, uncontrolled diabetes (HbA1c > 9%), thyroid disease, symptomatic heart disease, immunologic disease, and severe liver and kidney disease were excluded. *Intervention and comparison*: OM3-FA monotherapy vs. other triglyceride-lowering drugs, such as fibrates, ezetimibe, gemfibrozil, nicotinic acid, and bile acid sequestrants. *Outcomes*: Studies in which lipid profile’s mean and standard deviation were not reported or could not be obtained by formula transformation were excluded. *Study design*: Trials with cross-over design, observational studies, reviews, case reports, abstracts, and protocols were excluded.

### Data extraction

Two reviewers (YW and TL) extracted data independently. They settled through negotiation or consulted a third reviewer (QC) if there were any discrepancies. After searching the four databases, the software (Endnote X9) was used to remove duplicates. Qualified records were further extracted for relevant data. The specific extraction contents are as follows: first author, year of publication, the country where the study was conducted, study design, trial registration number, duration of treatment, the sample size of experimental group and control group, type and dose of OM3-FA, age, gender, and body mass index (BMI) of study subjects, the diet of the participants, reported endpoints. The data from the highest one was extracted for studies in which multiple dosages of OM3-FA were reported simultaneously. We emailed the corresponding author for more details on literature that lacked enough information. If no response was received, we only analyzed the available data.

### Risk of bias assessment

The risk of bias was assessed by two reviewers (YC and CL) independently based on the Cochrane collaboration tool (26). Any disagreements were settled by consensus or by the third reviewer. Each study was classified as high, unclear, or low risk of bias for seven aspects (random sequence generation, allocation concealment, blinding of participants and personnel, blinding of study outcome examination, selective reporting, completeness of study outcome information, and other sources of bias).

### Statistical analysis

Stata17.0 and Review Manager 5.3 software were used to perform the meta-analysis. Since all outcomes were continuous variables, we standardized the extracted data to obtain the mean and standard deviation. When the units of lipid levels reported as mmol/L, we uniformly converted mmol/L to mg/dl according to the unit conversion method (triglyceride: 1 mg/dL = 0.01129 mmol/L, cholesterol: 1 mg/dL = 0.02586 mmol/L). We calculated the mean and standard deviation using specific formulas for the studies reporting values with median and interquartile ranges (27, 28). Data presented as standard error (SE) were converted to standard deviation (SD) by the equation SD = SE × $\sqrt{n}$. When the literature data form was reported as Median (minimum-maximum) and mean with 95% confidence interval (CI) or the articles only described the mean and standard deviation at baseline and post-treatment, we used appropriate methods described in the Cochrane Handbook for Systematic Reviews of Interventions^3^ for transformation. Due to the differences in baseline characteristics among the included trials, a random-effect model was used to perform the meta-analysis. Heterogeneity among studies was evaluated by Cochran Q and I^2^ statistics, and the *p*-value of < 0.1 and *I*^2^ > 50% indicates significant heterogeneity. Besides, we performed subgroup analysis according to the intervention time (<12 weeks or ≥12 weeks), baseline TG levels (150–200, 200–500, and ≥500 mg/dl), and the dosage (<4 or ≥4 g/days) and kind (EPA, DHA, EPA+DHA) of OM3-FA. However, subgroup analyses based on fatty acid species were not performed for the combined therapy of OM3-FA and statins, because EPA and DHA were used in all studies except in one study that only used EPA. Sensitivity analysis was performed to check the robustness of the results. Funnel plot and Egger’s test were conducted to assess publication bias for outcomes involving ten or more studies. A *p*-value < 0.1 was defined as significant publication bias. Then, the trim-and-fill computation was used to estimate the effect of publication bias on interpreting the results. This meta-analysis expressed the results as mean difference (MD) and 95% CI, and the *p* < 0.05 was considered statistically significant.

## Results

### Search results

**FIGURE 1:**
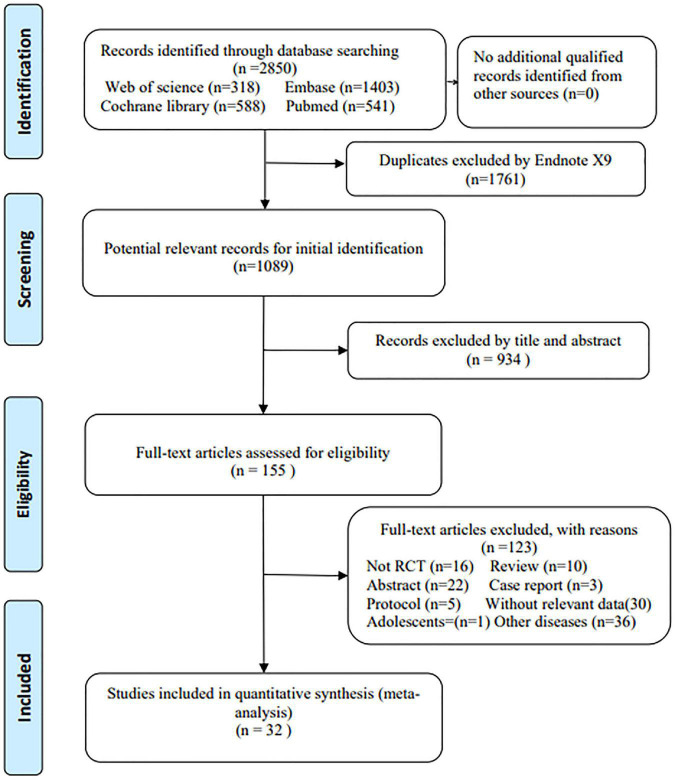
The flowchart of the study selection process.

A total of 2,850 papers were identified by searching four databases. No additional eligible articles were identified from other sources. Duplicate articles (*n* = 1761) were removed by Endnote X9, and another 1,089 articles were approved to be further screened. After reading titles and abstracts, 155 were left for full-text reading, and then 123 articles were excluded for various reasons. Finally, 32 studies with 15,903 participants were enrolled in this meta-analysis (11–14, 18–21, 23, 24, 29–50). The flowchart of study selection is shown in Figure 1.

### Characteristics of included studies

Among the 32 included studies, there were 16 multicenter randomized controlled trials (11, 12, 14, 18–21, 23, 24, 29, 38, 41–43, 45, 46). All RCTs were double-blind except for six trials (13, 31, 35, 46, 47, 49). The study duration varied from 4 to 48 weeks. The mean age of the participants ranged from 46.7 to 65 years. The mean BMI of the subjects ranged from 24.3 to 35.2 kg/m^2^. The dosage of EPA/DHA ranged from 1.24 to 4 g/days. The dosage of EPA was 0.6–4 g/days. The dosage of DHA was 3–4 g/days. The trials were conducted in the following regions: Worldwide (*n* = 4), USA (*n* = 9), Korea (*n* = 6), Australia (*n* = 5), Iran (*n* = 2), India (*n* = 1), German (*n* = 1), UK (*n* = 1), Japan (*n* = 1), Norway (*n* = 1), and China (*n* = 1). These studies were published between 1993 and 2022. The mean baseline TG level in the experimental group ranged from 154.2 to 699 mg/dl. The placebo controls used in most studies were corn oil and olive oil. The detailed characteristics of included studies are summarized in Table 1.

**TABLE 1:** Characteristics of included studies.

References	Registration	Design	Location	Duration	Group	Sample (n)	Male	Age (Year) mean (SD)	BMI (kg/m^2^) Mean (SD)	Endpoints	Mean baseline TG level (mg/dl)	Diet
Bays et al. (29)	NCT01047683	RCT M D	Worldwide	12-week	Placebo※ EPA 4 g/days	76 76	76% 77%	53.4 (8.3) 51.9 (10.3)	31.0 (4.3) 30.4 (4.3)	TG, TC, HDL-C, LDL-C, VLDL-C, non-HDL-C, Apo-B	703.0 679.5	Maintain the National Cholesterol Education Program Therapeutic Lifestyle Changes Diet
Bays et al. (11)	NCT01893515	RCT M D	USA	12-week	Placebo (Miglyol 812) EPA 0.6 g/days	43 41	71% 67%	51.6 (11.4) 53.5 (8.8)	32.3 (4.6) 31.7 (4.4)	TG, TC, HDL-C, LDL-C, VLDL-C, non-HDL-C, Apo-B, Apo-AI	727.4 681.3	Maintain the National Cholesterol Education Program Therapeutic Lifestyle Changes diet
Chan et al. (30)	NR	RCT D	Australia	6-week	Placebo (corn coil) EPA/DHA 4 g/days Atorvastatin 40 mg/days Atorvastatin 40 mg + EPA/DHA 4 g/days	13 13 14 12	NR	50 (2.5) 58 (2.2) 52 (2.7) 53 (2.6)	32.4 (0.8) 35.2 (1.2) 34.5 (1.3) 32.5 (0.8)	TG, TC, HDL-C, LDL-C, non-HDL-C, Apo-B, Apo-AI	265.8 177.2 168.3 177.2	Continue their habitual diet and to keep physical exercise constant
Contacos et al. (31)	NR	RCT S	Australia	6-week	Placebo^※^ EPA/DHA 3 g/days	11 10	90% 36%	64 (7.0) 54 (11.0)	24.9 (2.3) 27.1 (2.7)	TG, TC, HDL-C, LDL-C, Apo-B, Apo-AI	425.3 407.6	NR
Davidson et al. (32)	NR	RCT D	USA	6-week	Placebo (vegetable oil) DHA 3 g/days	8 9	50% 89%	57 (11.3) 58 (12.0)	28.5 (5.1) 27.8 (3.0)	TG, HDL-C, LDL-C, non-HDL-C	236.0 296.0	Keep an National Cholesterol Education Program Step I diet
Kastelein et al. (18)	NCT01242527	RCT M D	Worldwide	12-week	Placebo (olive oil) EPA/DHA 4 g/days	99 99	78% 72%	50.8 (10.6) 52.9 (10.9)	30.4 (4.3) 31.0 (5.1)	TG, TC, LDL-C, HDL-C, non-HDL-C, VLDL-C, Apo-B, Apo-AI	682.0 655.0	Follow the National Cholesterol Education Program Therapeutic Lifestyle Changes (TLC) diet
Kelley et al. (33)	NR	RCT D	USA	14-week	Placebo (olive oil) DHA 3 g/days	17 17	NR	53.1 (4.1) 55.0 (8.2)	30.6 (3.3) 27.8 (2.9)	TG, TC, LDL-C, HDL-C	240.1 225.9	Continue regular diets and activity levels throughout the study.
Khandelwal et al. (34)	ETH 2006/N46	RCT D	India	4-week	Placebo (safflower-seed oil) EPA/DHA 2 g/days	46 40	89% 95%	46.1 (6.1) 48.2 (5.7)	24.3 (3.4) 25.7 (3.8)	TG, TC, LDL-C, HDL-C, Apo-B, Apo-AI	140.9 165.7	Maintain habitual diet and lifestyle
Koh et al. (35)	NR	RCT S	Korea	8-week	placebo^※^ EPA/DHA 2 g/days	49 50	59% 56%	54 (7.0) 55 (7.1)	25.1 (2.3) 25.5 (2.5)	TG, TC, LDL-C, HDL-C, non-HDL-C, Apo-B, Apo-AI	267.0 290.0	Maintain a low fat diet
Maki et al. (12)	NR	RCT M D	USA	8-week	Placebo (olive oil) EPA 2 g/days	53 52	62% 52%	54.4 (10.1) 49.1 (11.8)	30.6 (5.1) 31.7 (5.8)	TG, TC, LDL-C, HDL-C, non-HDL-C	251.5 236.8	Maintain consistent dietary habits
Mori et al. (36)	NR	RCT D	Australia	6-week	placebo (olive oil) EPA 4 g/days DHA 4 g/days	20 19 17	NR	48.4 (8.9) 48.9 (7.4) 49.1 (9.1)	28.4 (2.2) 29.0 (3.1) 28.9 (2.9)	TG, TC, LDL-C, HDL-C	180.7 178.1 199.4	Maintain their usual diets, physical activities, lifestyle
Mozaffarian et al. (23)^¶^	NCT03398005 NCT03361501	RCT M D	Worldwide	26-week	Placebo (cornstarch) EPA/DHA 1.24 g/days	148 372	68% 64%	53.9 (11.8) 55.3 (10.9)	31.5 (5.5) 31.5 (5.1)	TG, LDL-C, HDL-C, non-HDL-C, VLDL-C	706.0 699.0	Maintain the National Cholesterol Education Program Therapeutic Lifestyle Changes Diet
Nicholls et al. (19)	NCT02104817	RCT M D	worldwide	48-week	Placebo (corn oil) EPA/DHA 4 g/days	6539 6539	65% 65%	62.5 (9.0) 62.5 (9.0)	32.2 (5.6) 32.2 (5.7)	TG, TC, LDL-C, HDL-C, non-HDL, Apo-B	246.4 247.0	Maintain stable diet
Oelrich et al. (37)	NR	RCT D	USA	12-week	Placebo (soy oil) EPA/DHA 4 g/days	15 42	93% 71%	52 (10.0) §	27 (3.0) 27 (4.0)	TG, LDL-C	230.4 248.1	Maintain weight and habitual dietary and exercise habits
Oh et al. (13)	NR	RCT S	Korea	8-week	Placebo^※^ EPA/DHA 4 g/days	42 44	55% 52%	54 (9.0) 55 (8.0)	26.50 (2.7) 26.18 (3.2)	TG, TC, HDL-C, LDL-C, non-HDL-C, Apo-B, Apo-AI	281.0 287.0	Maintain a low fat diet
Schuchardt et al. (38)	NR	RCT M D	German	24-week	Placebo (corn oil) EPA/DHA 1.68 g/days	35 39	34% 56%	62 (8.2) 61.6 (7.5)	26 (3.3) 25.8 (3.0)	TG, TC, HDL-C, LDL-C	133.8 154.2	Maintain their usual exercise and dietary habits
Shidfar et al. (39)	NR	RCT D	Iran	10-week	Placebo (linoleic acid) EPA/DHA 2 g/days	25 25	48% 48%	54.1 (11.1) 53.4 (11.7)	29.0 (0.7) 28.4 (0.5)	TG, TC, HDL-C, LDL-C, Apo-AI, Apo-B	306.4 299.2	Maintain their usual diet and physical activity level
Shidfar et al. (40)	NR	RCT D	Iran	10-week	Placebo (linoleic acid) EPA 1 g/days	19 16	37% 31%	54.4 (12.2) 54.4 (11.7)	27.6 (3.0) 26.9 (2.2)	TG, TC, HDL-C, LDL-C, Apo-AI, Apo-B	311.5 304.0	Maintain their usual diets, physical activities, and lifestyle
Su et al. (14)	NCT017256	RCT M D	China	8-week	Placebo (olive oil) EPA/DHA 4 g/days	87 84	74% 66%	54.4 (10.7) 53.7 (11.0)	26.7 (3.9) 26.6 (3.7)	TG, TC, HDL-C, LDL-C, non-HDL-C	336.9 351.2	Keep a low fat diets
Ballantyne et al. (41)	NCT01047501	RCT M D	USA	12-week	statin+Placebo^※^ statin+EPA 4 g/days	233 233	62% 61%	61.2 (10.1) 61.1 (10.0)	33.0 (5.0) 32.7 (5.0)	TG, TC, HDL-C, LDL-C, non-HDL-C, VLDL-C, Apo-B	259.0 264.8	Maintain stable diet and exercise
Bays et al. (42)	NCT00435045	RCT M D	USA	16-week	Atorvastatin+Placebo (corn oil) Atorvastatin+EPA/DHA 4 g/days	122 123	58% 58%	56 (10.8) 56.3 (9.6)	31.0 (4.0) 30.2 (4.6)	TG, TC, HDL-C, LDL-C, non-HDL-C, VLDL-C, Apo-B, Apo-A1	339.0 345.5	Follow the National Cholesterol Education Program therapeutic lifestyle changes diet
Davidson et al. (43)	NCT00246701	RCT M D	USA	8-Week	Simvastatin 40 mg/days+Placebo (vegetable oil) Simvastatin 40 mg+EPA/DHA 4 g/days	132 122	61% 54%	59.3 (10.8) 60.3 (10.1)	31.5 (5.5) 31.0 (5.4)	TG, TC, LDL-C, HDL-C, VLDL-C, non-HDL-C, Apo-B	286.7 282.0	Participants received dietary counseling on the Therapeutic Lifestyle Changes diet
Durringt on et al. (44)	NR	RCT D	UK	24-week	Simvastatin+Placebo (corn oil) Simvastatin+EPA/DHA 4 g/days	29 30	69% 77%	54.8 (10.2) 55.2 (7.0)	28.4 (4.2) 28.8 (2.8)	TG, TC, HDL-C, LDL-C, VLDL-C, Apo-B, Apo-AI	407.6 336.7	Patients received dietary advice and maintained their diet unchanged throughout the trial
Jun et al. (20)	NCT03482180	RCT M D	Korea	8-week	Atorvastatin 20 mg+placebo (olive oil) Atorvastatin 20 mg+EPA/DHA 4 g/days	103 97	63% 66%	58.0 (11.4) 58.7 (10.1)	27.0 (3.4) 27.3 (3.5)	TG, TC, VLDL-C, LDL-C, HDL-C, non-HDL-C, Apo-B, Apo-AI	293.0 298.5	NR
Kim et al. (45)	NCT03026933	RCT M D	Korea	8-week	Rosuvastatin 20 mg/days+placebo^※^ Rosuvastatin 20 mg/days+EPA/DHA 4 g/days	104 97	64% 61%	56.6 (10.5) 59.7 (10.8)	27.6 (3.6) 27.4 (3.7)	TG, TC, VLDL-C, LDL-C, HDL-C, non-HDL-C, Apo-B, Apo-AI	279.6 284.0	NR
Lee et al. (46)	NR	RCT O M	Korea	8-week	Statin+no placebo Statin+EPA/DHA 4 g/days	17 17	NR	56§(mean)	25.7§(mean)	TG, TC, LDL-C, HDL-C, Apo-B, Apo-AI	321.5 295.7	NR
Maki et al. (21)	NCT01408303	RCT M D	USA	6-week	Statin+placebo (olive oil) Statin+EPA/DHA 4 g/days	216 216	57% 63%	61.5 (9.6) 60.1 (9.2)	32.7 (5.3) 33.3 (6.6)	TG, TC, VLDL-C, LDL-C, HDL-C, non-HDL-C, Apo-B, Apo-AI	280.0 287.0	Maintain stable diet
Meyer et al. (47)	NR	RCT	Australia	24-week	Statin+Placebo (olive oil) Statin+EPA/DHA 2.72 g/days	15 15	67% 67%	59 (7.7) 53 (19.4)	27.9 (4.6) 26.2 (0.8)	TG, TC, HDL-C, LDL-C	179.0 209.1	NR
Ng et al. (48)	NR	RCT D	Australia	6-week	Atorvastatin+Placebo (corn oil) Atorvastatin40 mg+EPA/DHA 4 g/days	13 11	NR	52 (10.8) 54 (6.6)	34.5 (5.0) 32.5 (3.0)	TG, TC, LDL-C, HDL-C, non-HDL-C, Apo-B, Apo-AI	166.6 181.6	NR
Nomura et al. (49)	NR	RCT	Japan	24-week	Pitavastatin 2 mg+no Placebo Pitavastatin 2 mg+EPA 1.8 g/days	64 72	53%	65 (3.0)§	27.3 (3.9)§	TC, TG, HDL-C, LDL-C	198.0 248.0 258.0	NR
Nordøy et al. (50)	NR	RCT D	Norway	5-week	Simvastatin 20 mg+placebo (corn oil) Simvastatin 20 mg+EPA/DHA 4 g/days	20 21	70% 71%	46.7 (7.8) 46.8 (9.2)	28.8 (3.7) 27.6 (4.0)	TG, TC, HDL-C, Apo-B, Apo-AI	268.5 244.5	Follow guidelines aiming at a dietary composition
Woo et al. (24)	NR	RCT M D	Korea	8-week	Atorvastatin 40 mg+placebo (UI-018) atorvastatin 40 mg+EPA 4 g/days	99 101	74% 88%	60.0 59.0 (mean)	25.5 (2.9) 26.8 (2.6)	TG, TC, VLDL-C, LDL-C, HDL-C, non-HDL-C, Apo-B, Apo AI	297.1 294.2	NR

NR, not report; RCT, randomized controlled trial; D, double-blinded; S, single-blinded; M, multicenter; O, open-label.

¶: The study pooled the results of two identical trials.

※: The type of placebo is unknown.

§: These values cover all participants.

### Risk of bias in included studies

**FIGURE 2:**
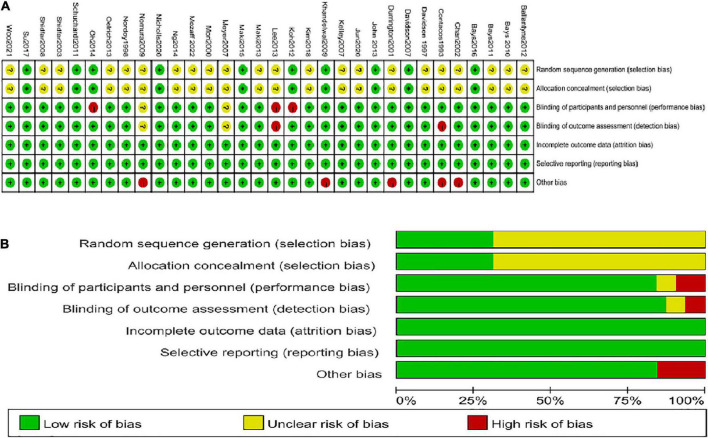
Summary of quality evaluation based on the Cochrane’s Risk of Bias Tool. **(A)** Risk of bias summary for each risk of bias item for each included study; **(B)** Risk of bias graph for each risk of bias item presented as percentages across all included studies.

Regarding selection bias, ten studies (11–14, 18, 19, 34, 35, 38, 43) were determined as “low risk” because they reported their specific randomization strategies and allocation concealment method. The remaining articles only described random assignment but did not point out the specific random sequence method and how to realize allocation concealment. Therefore, they were identified as “unclear risk” Concerning performance bias, 26 studies used a double-blind design, which was identified as “low risk” and three studies (13, 31, 35) used a single-blind method, which was also identified as “low risk” according to the corresponding aspect. Two articles (47, 49) were considered “unclear risk” because of not specifying whether blinding was used. Only one study (46) was deemed “high risk” because of the open-label design. Most studies registered for the clinical trial protocol, and even those not registered reported all the expected results. There were a few trials (13, 20, 35, 45) with patients dropping out, but the number was balanced between the experimental and control groups, and not enough affected the results. Besides, no factors were found that could affect the test results in all studies. Hence, total studies were identified as “low risk” for the detection bias, follow-up bias, and reporting bias. However, several studies (30, 31, 34, 44, 49) in which differences in baseline characteristics were significant between the experimental group and control group could be considered as “high risk” for the other bias. The summary and graph are shown in Figure 2.

### The effect of omega-3 fatty acids on triglyceride

**FIGURE 3:**
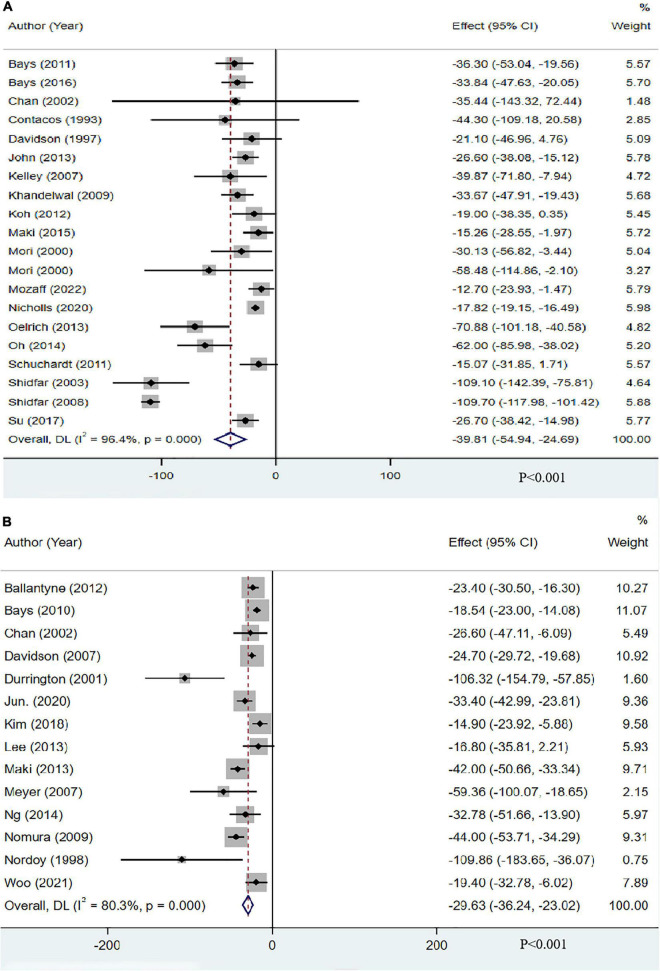
The effect of OM3-FA on TG. **(A)** OM3-FA monotherapy; **(B)** Combined therapy of statins plus OM3-FA.

Nineteen studies (20 groups) with 13,612 participants reported the effect of OM3-FA on TG. The pooled analysis showed that OM3-FA exerted a significant reduction in TG concentrations compared to placebo (MD: −39.81, 95% CI: −54.94 to −24.69; *p* < 0.001), but significant heterogeneity was identified (*I*^2^ = 96.4%, *p*_*he*_ < 0.001) (Figure 3A). Fourteen studies with 2291 individuals reported the effect of OM3-FA combined with statins on TG. Similar effect was identified (MD: −29.63, 95% CI: −36.24 to −23.02; *p* < 0.001), which also accompanied by obvious heterogeneity (*I*^2^ = 80.3%, *p*_*he*_ < 0.001) (Figure 3B). All subgroup analyses showed that OM3-FA effectively reduced TG, regardless of whether OM3-FA monotherapy or combined therapy of statins plus OM3-FA (Tables 2, 3).

**TABLE 2:** Subgroup analysis of the effect of OM3-FA monotherapy on lipid profile.

Serum lipids	Subgroup type	Group	Study (*n*)	Sample (*n*)	Effect estimate	*P*-value	*I* ^2^	P_*heterogeneity*_
TG	Dosage	<4 g	11	1122	−40.74 [−68.40, −13.08]	0.004	97%	<0.00001
		≥4 g	8	12490	−35.03 [−46.26, −23.80]	<0.00001	78%	<0.0001
	type	EPA	5	411	−41.19 [−62.70, −19.69]	0.0002	85%	<0.0001
		DHA	3	88	−31.91 [−50.84, −12.98]	0.001	0%	0.41
		EPA+DHA	12	13113	−39.17 [−60.95, −17.39]	0.0004	98%	<0.00001
	Baseline TG level (mg/dl)	<200	4	262	−27.54 [−37.53, −17.54]	<0.00001	1%	0.40
		≥200, <500	11	12398	−48.26 [−75.02, −21.51]	0.0004	98%	<0.00001
		≥500	4	952	−26.43 [−37.12, −15.75]	<0.00001	63%	0.05
	Duration (Week)	<12	11	767	−47.22 [−74.56, −19.88]	0.0007	96%	<0.00001
		≥12	8	12845	−26.56 [−34.99, −18.13]	<0.00001	73%	0.0005
TC	Dosage	<4 g	9	585	−0.67 [−5.01, 3.66]	0.76	49%	0.05
		≥4 g	7	12433	−4.72 [−8.70, −0.73]	0.02	72%	0.0007
	type	EPA	5	411	−2.71 [−10.45, 5.03]	0.49	77%	0.002
		DHA	2	71	14.78 [4.61, 24.95]	0.004	0%	0.82
		EPA+DHA	10	12536	−4.55 [−6.95, −2.15]	<0.0002	36%	0.12
	Baseline TG level (mg/dl)	<200	4	262	2.97 [−5.91, 11.86]	0.51	63%	0.03
		≥200, <500	9	12324	−2.31 [−4.99, 0.38]	0.09	48%	0.05
		≥500	3	432	−10.01 [−15.52, −4.50]	0.0004	51%	0.13
	Duration (Week)	<12	10	750	−1.57 [−5.25, 2.11]	0.40	47%	0.04
		≥12	6	12268	−4.27 [−9.65, 1.10]	0.12	79%	0.0003
HDL-C	Dosage	<4 g	11	1122	2.01 [−0.94, 4.96]	0.18	67%	0.0008
		≥4 g	7	12433	1.32 [−0.37, 3.01]	0.12	35%	0.15
	type	EPA	5	411	3.64 [−3.01, 10.28]	0.28	85%	<0.00001
		DHA	3	88	3.59 [−0.53, 7.71]	0.09	32%	0.23
		EPA+DHA	11	13056	1.22 [0.70, 1.73]	<0.00001	0%	0.73
	Baseline TG level (mg/dl)	<200	4	262	2.94 [0.67, 5.21]	0.01	0%	0.46
		≥200, <500	10	12341	1.16 [0.64, 1.68]	<0.00001	0%	0.71
		≥500	4	952	5.21 [−3.76, 14.18]	0.25	87%	<0.0001
	Duration (Week)	<12	11	767	0.53 [−0.83, 1.90]	0.44	0%	0.46
		≥12	7	12788	3.75 [−0.42, 7.92]	0.08	79%	<0.0001
LDL-C	Dosage	<4 g	11	1122	13.46 [3.65, 23.26]	0.007	81%	<0.00001
		≥4 g	8	12490	6.67 [1.31, 12.04]	0.01	58%	0.01
	type	EPA	5	411	24.05 [3.84, 44.26]	0.02	91%	<0.00001
		DHA	3	88	16.91 [8.76, 25.06]	<0.0001	0%	0.96
		EPA+DHA	12	13113	4.39 [0.22, 8.55]	0.04	40%	0.07
	Baseline TG level (mg/dl)	<200	4	262	3.81 [−4.80, 12.42]	0.39	38%	0.16
		≥200, <500	11	12398	9.86 [3.25, 16.48]	0.003	81%	<0.00001
		≥500	14	952	13.42 [0.05, 26.79]	0.05	71%	0.01
	Duration (Week)	<12	11	767	9.31 [1.27, 17.34]	0.02	78%	<0.00001
		≥12	8	12845	10.09 [2.54, 17.65]	0.009	71%	0.001
VLDL-C	Dosage	<4 g	2	604	−23.00 [−50.94, 4.94]	0.11	85%	0.009
		≥4 g	2	348	−26.40 [−35.72, −17.09]	<0.00001	0%	0.45
	type	EPA	2	235	−35.84 [−48.49, −23.18]	<0.00001	0%	0.74
		DHA	0	–	–	–	–	–
		EPA+DHA	2	717	−17.46 [−32.50, −2.43]	0.02	67%	0.08
	Baseline TG level (mg/dl)	<200	0	–	–	–	–	–
		≥200, <500	0	–	–	–	–	–
		≥500	4	952	−25.12 [−37.09, −13.14]	<0.0001	62%	0.05
	Duration (Week)	<12	0	–	–	–	–	–
		≥12	4	952	−25.12 [−37.09, −13.14]	<0.0001	62%	0.05
Non-HDL-C	Dosage	<4 g	5	822	−2.85 [−7.63, 1.93]	0.24	62%	0.03
		≥4 g	6	12357	−7.32 [−10.67, −3.96]	<0.0001	55%	0.05
	type	EPA	3	337	−5.93 [−16.20, 4.35]	0.26	86%	0.0009
		DHA	1	17	4.30 [−5.78, 14.38]	0.40	–	–
		EPA+DHA	7	12825	−5.41 [−6.56, −4.25]	<0.00001	4%	0.39
	Baseline TG level (mg/dl)	<200	1	26	−11.99 [−24.97, 0.99]	0.07	–	–
		≥200, <500	6	12201	−3.04 [−6.26, 0.18]	0.06	64%	0.02
		≥500	4	952	−9.39 [−13.84, −4.94]	< 0.0001	39%	0.18
	Duration (Week)	<12	6	499	−2.92 [−7.20, 1.36]	0.18	55%	0.05
		≥12	5	12680	−7.83 [−11.48, −4.18]	<0.0001	59%	0.04
Apo-B	Dosage	<4 g	6	375	−4.11 [−10.44, 2.21]	0.20	37%	0.16
		≥4 g	5	12188	−2.02 [−5.65, 1.61]	0.28	48%	0.11
	type	EPA	3	270	−5.93 [−16.20, 4.35]	0.41	61%	0.08
		DHA	0	–	–	–	–	–
		EPA+DHA	8	12293	−5.41 [−6.56, −4.25]	0.26	26%	0.22
	Baseline TG level (mg/dl)	<200	2	112	−3.63 [−17.16, 9.91]	0.60	0%	0.74
		≥200, <500	6	12019	−3.55 [−7.84, 0.74]	0.10	40%	0.14
		≥500	3	432	−1.01 [−8.31, 6.30]	0.79	73%	0.02
	Duration (Week)	<12	7	403	−5.19 [−9.70, −0.69]	0.02	0%	0.58
		≥12	4	12160	−0.86 [−4.80, 3.07]	0.67	61%	0.05
Apo-AI	Dosage	<4 g	6	375	1.79 [−2.76, 6.33]	0.44	31%	0.20
		≥4 g	3	309	−3.20 [−8.79, 2.39]	0.26	72%	0.03
	type	EPA	2	119	4.62 [−0.08, 9.31]	0.05	0%	0.56
		DHA	0	–	–	–	–	–
		EPA+DHA	7	565	−2.30 [−6.10, 1.49]	0.23	49%	0.06
	Baseline TG level (mg/dl)	<200	2	112	4.72 [−2.90, 12.34]	0.23	0%	0.60
		≥200, <500	5	291	−1.40 [−5.98, 3.18]	0.55	39%	0.16
		≥500	2	281	−1.40 [−12.18, 9.37]	0.80	92%	0.0006
	Duration (Week)	<12	7	403	−0.14 [−4.49, 4.21]	0.95	40%	0.12
		≥12	2	281	−1.40 [−12.18, 9.37]	0.80	92%	0.0006

**TABLE 3:** Subgroup analysis of the effect of combined therapy of statins plus OM3-FA on lipid profile.

Serum lipids	Subgroup type	Group	Study (*n*)	Sample (*n*)	Effect estimate	*P*-value	*I* ^2^	P_*heterogeneity*_
TG	Dosage	<4 g	2	166	−44.83 [−54.27, −35.38]	<0.00001	0%	0.47
		≥4 g	12	2125	−27.04 [−33.40, −20.67]	<0.00001	77%	<0.00001
	Baseline TG level (mg/dl)	<200	3	80	−33.01 [−46.16, −19.87]	<0.00001	0%	0.37
		≥200, <500	11	2211	−28.93 [−36.16, −21.69]	<0.00001	84%	<0.00001
	Duration (Week)	<12	9	1398	−27.51 [−35.11, −19.92]	<0.00001	72%	0.0004
		≥12	5	893	−36.55 [−51.24, −21.86]	<0.00001	89%	<0.00001
TC	Dosage	<4 g	2	166	−11.69 [−23.72, 0.34]	0.06	55%	0.14
		≥4 g	12	2125	−6.53 [−9.04, −4.03]	<0.00001	69%	0.0002
	Baseline TG level (mg/dl)	<200	3	80	−10.91 [−20.69, −1.12]	0.03	12%	0.32
		≥200, <500	11	2211	−6.61 [−9.11, −4.11]	<0.00001	72%	0.0001
	Duration (Week)	<12	9	1398	−4.98 [−6.78, −3.18]	<0.00001	18%	0.29
		≥12	5	893	−11.18 [−17.77, −4.60]	0.0009	85%	<0.0001
HDL-C	Dosage	<4 g	2	166	1.19 [−3.18, 5.57]	0.59	0%	0.87
		≥4 g	12	2125	0.90 [−1.73, 3.52]	0.50	75%	<0.00001
	Baseline TG level (mg/dl)	<200	3	80	3.40 [−2.49, 9.28]	0.26	0%	0.93
		≥200, <500	11	2211	0.67 [−1.91, 3.25]	0.61	77%	<0.00001
	Duration (Week)	<12	9	1398	2.33 [0.31, 4.36]	0.02	44%	0.07
		≥12	5	893	−1.76 [−7.47, 3.95]	0.55	83%	<0.0001
LDL-C	Dosage	<4 g	2	166	−1.23 [−9.09, 6.62]	0.76	0%	0.40
		≥4 g	11	2082	−0.83 [−4.20, 2.54]	0.63	56%	0.01
	Baseline TG level (mg/dl)	<200	3	80	−1.12 [−13.87, 11.63]	0.86	0%	0.63
		≥200, <500	10	2168	−0.92 [−4.24, 2.39]	0.58	60%	0.007
	Duration (Week)	<12	8	1357	0.07 [−3.62, 3.77]	0.97	44%	0.09
		≥12	5	891	−2.61 [−8.61, 3.40]	0.40	59%	0.04
VLDL-C	Dosage	<4 g	0	–	–	–	–	–
		≥4 g	8	1998	−20.13 [−24.76, −15.50]	<0.00001	53%	0.04
	Baseline TG level (mg/dl)	<200	0	–	–	–	–	–
		≥200, <500	8	1998	−20.13 [−24.76, −15.50]	<0.00001	53%	0.04
	Duration (Week)	<12	5	1273	−21.41 [−28.17, −14.65]	<0.00001	61%	0.04
		≥12	3	725	−18.70 [−26.45, −10.96]	<0.00001	56%	0.10
Non-HDL-C	Dosage	<4 g	0	–	–	–	–	–
		≥4 g	9	1995	−8.71 [−11.45, −5.98]	<0.00001	59%	0.01
	Baseline TG level (mg/dl)	<200	2	50	−7.74 [−18.08, 2.60]	0.14	0%	1.00
		≥200, <500	7	1945	−8.82 [−11.83, −5.82]	<0.00001	69%	0.003
	Duration (Week)	<12	7	1323	−8.08 [−10.34, −5.82]	<0.00001	9%	0.36
		≥12	2	672	−9.90 [−19.09, −0.71]	0.03	92%	0.0003
Apo-B	Dosage	<4 g	0	–	–	–	–	–
		≥4 g	12	2108	−3.50 [−5.37, −1.64]	0.0002	32%	0.13
	Baseline TG level (mg/dl)	<200	2	50	−7.51 [−18.18, 3.16]	0.17	0%	0.93
		≥200, <500	10	2058	−3.37 [−5.38, −1.36]	0.001	42%	0.08
	Duration (Week)	<12	9	1398	−2.61 [−4.25, −0.96]	0.002	0%	0.73
		≥12	3	710	−5.59 [−11.52, 0.34]	0.06	76%	0.02
Apo-AI	Dosage	<4 g	0	–	–	–	–	–
		≥4 g	10	1418	−2.01 [−3.07, −0.95]	0.0002	0%	0.53
	Baseline TG level (mg/dl)	<200	2	50	3.00 [−5.68, 11.68]	0.50	0%	1.00
		≥200, <500	8	1368	−2.09 [−3.16, −1.02]	0.0001	0%	0.46
	Duration	<12	8	1144	−1.78 [−2.94, −0.61]	0.003	0%	0.65
		≥12	2	274	−3.56 [−7.50, 0.37]	0.08	51%	0.15

### The effect of omega-3 fatty acids on total cholesterol

**FIGURE 4:**
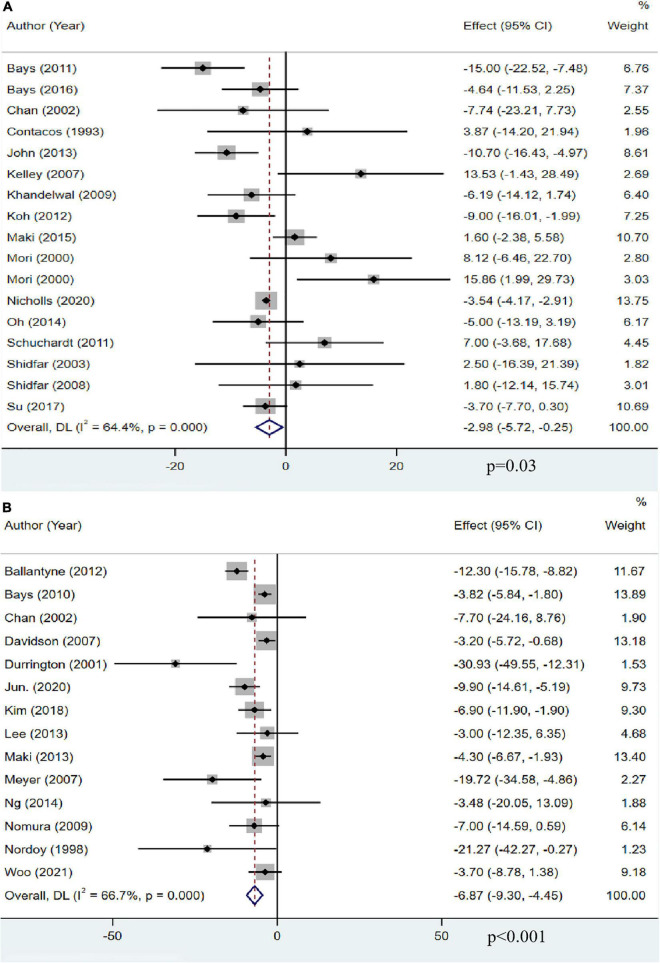
The effect of OM3-FA on TC. **(A)** OM3-FA monotherapy; **(B)** Combined therapy of statins plus OM3-FA.

Sixteen studies with 13,018 participants reported the effect of OM3-FA on TC. The overall analysis showed that OM3-FA significantly reduced TC level (MD: −2.98, 95% CI: −5.72 to −0.25, *p* = 0.03; *I*^2^ = 64.4%, *p*_*he*_ < 0.001) (Figure 4A). Fourteen studies with 2,291 patients reported the effect of OM3-FA added to statins on TG. The combined result showed that the TC reduction was more significant (MD: −6.87, 95% CI: −9.30 to −4.45, *p* < 0.001; *I*^2^ = 66.7%, *p*_*he*_ < 0.001) (Figure 4B). In subgroup analyses, with respect to OM3-FA monotherapy, we found that the TC reduction was statistically significant only when the dose of OM3-FA was ≥4 g (MD: −4.72, 95% CI: −8.70 to −0.73, *p* = 0.02; *I*^2^ = 72%, *p*_*he*_ = 0.007), the baseline TG level of participants was ≥500 mg/dL (MD: −10.01, 95% CI: −15.52 to −4.50, *p* = 0.0004; *I*^2^ = 51%, *p*_*he*_ = 0.13), EPA and DHA were used together (MD: −4.55, 95% CI: −6.95 to −2.15, *p* < 0.0002; *I*^2^ = 36%, *p*_*he*_ = 0.12). However, when DHA was used alone, it increased the level of TC (MD: 14.78, 95% CI: 4.61 to 24.95, *p* = 0.004; *I*^2^ = 0%, *p*_*he*_ = 0.82) (Table 2). Regarding the combined therapy of statins plus OM3-FA, dose-based subgroup analysis showed that the TC reduction was not significant if the dose was <4 g (MD: −11.69, 95% CI: −23.72 to 0.34, *p* = 0.06; *I*^2^ = 55%, *p*_*he*_ = 0.14) (Table 3).

### The effect of omega-3 fatty acids on high-density lipoprotein cholesterol

**FIGURE 5:**
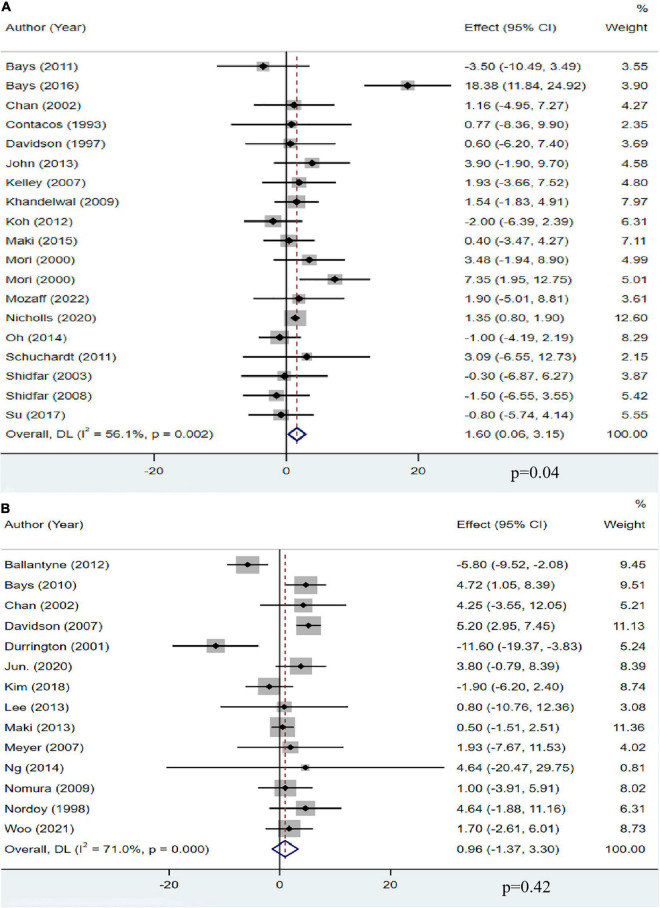
The effect of OM3-FA on HDL-C. **(A)** OM3-FA monotherapy; **(B)** Combined therapy of statins plus OM3-FA.

A total of 18 studies, including 13,555 participants, investigated the effect of OM3-FA on HDL-C. The pooled analysis showed that OM3-FA increased the concentration of HDL-C compared with placebo (MD: 1.60, 95% CI: 0.06 to 3.15; *p* = 0.04), with significant heterogeneity (*I*^2^ = 56.1%, *p*_*he*_ = 0.002) (Figure 5A). However, by removing one article (11) which favored that OM3-FA significantly increased HDL-C levels, no significant heterogeneity was identified (*I*^2^ = 0%, *p*_*he*_ = 0.51). Fourteen studies with 2,291 patients assessed the effect of OM3-FA added to statins on HDL-C, and the pooled result demonstrated that no significant impact was identified (MD: 0.96, 95% CI: −1.37 to 3.30; *p* = 0.42), with apparent heterogeneity (*I*^2^ = 71%, *p*_*he*_ < 0.001) (Figure 5B). By removing two articles that favored that OM3-FA reduced HDL-C levels (41, 44), the pooled result was reversed (MD: 2.50, 95% CI: 0.90 to 4.10, *p* = 0.002), without significant heterogeneity (*I*^2^ = 32%, *p*_*he*_ = 0.13). In the subgroup analyses, we found that OM3-FA monotherapy significantly increased HDL-C levels only in the case of EPA and DHA used together (MD: 1.22, 95% CI: 0.70 to 1.73, *p* < 0.00001; *I*^2^ = 0%, *p*_*he*_ = 0.73) and participants with a baseline TG level less than 500 mg/dL (Table 2). When OM3-FA was used with statins, subgroup analysis showed that the HDL-C level increased when the intervention duration was <12 weeks (MD: 2.33, 95% CI: 0.31 to 4.36, *p* = 0.02; *I*^2^ = 44%, *p*_*he*_ = 0.07) (Table 3).

### The effect of omega-3 fatty acids on low-density lipoprotein cholesterol

**FIGURE 6:**
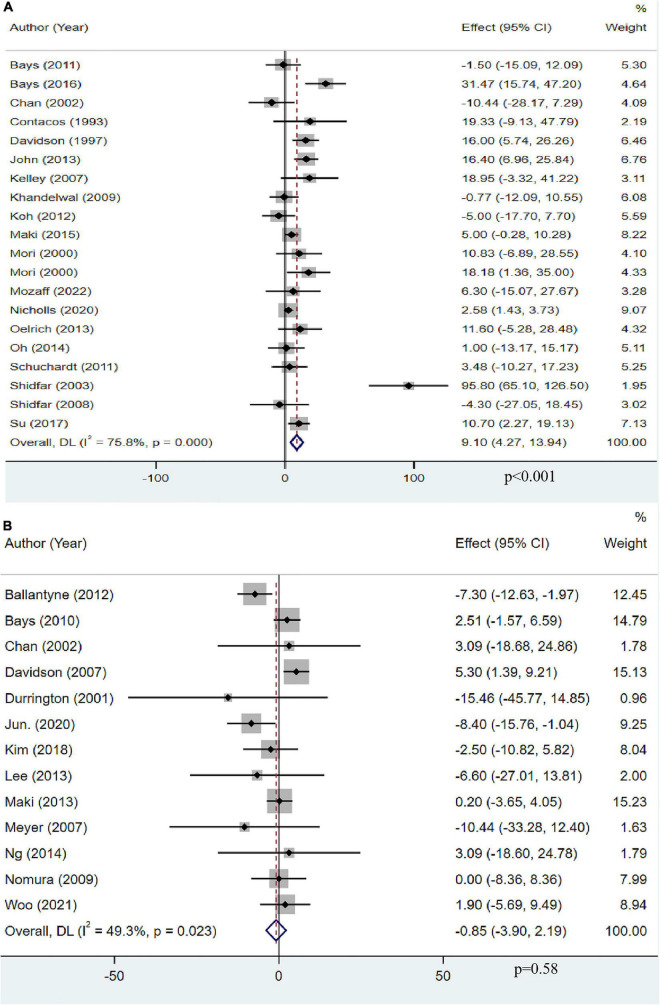
The effect of OM3-FA on LDL-C. **(A)** OM3-FA monotherapy; **(B)** Combined therapy of statins plus OM3-FA.

A total of 19 studies with 13,612 objects described the effect of OM3-FA on LDL-C. The pooled result showed that OM3-FA significantly increased LDL-C levels compared to the control group (MD: 9.10, 95% CI: 4.27 to 13.94; *p* < 0.001), with large heterogeneity (*I*^2^ = 75.8%, *p*_*he*_ < 0.001) (Figure 6A). Thirteen studies with 2,248 patients assessed the effect of OM3-FA in combination with statins on LDL-C. However, the level of LDL-C was not increased compared to the control group (MD: −0.85, 95% CI: −3.90 to 2.19, *p* = 0.58; *I*^2^ = 49.3%, *p*_*he*_ = 0.023), which is quite different from the result when OM3-FA was used alone (Figure 6B). In subgroup analyses, we found that OM3-FA had no significant effect on LDL-C if the participants had a baseline TG level of <200 mg/dL (MD: 3.81, 95% CI: −4.80 to 12.42, *p* = 0.39; *I*^2^ = 38%, *p*_*he*_ = 0.16) or ≥500 mg/dL (MD: 13.42, 95% CI: 0.05 to 26.79, *p* = 0.05; *I*^2^ = 71%, *p*_*he*_ = 0.01) (Table 2). None of the subgroups demonstrated an increase in LDL-C levels for the combined therapy of OM3-FA and statins (Table 3).

### The effect of omega-3 fatty acids on very low-density lipoprotein cholesterol

**FIGURE 7:**
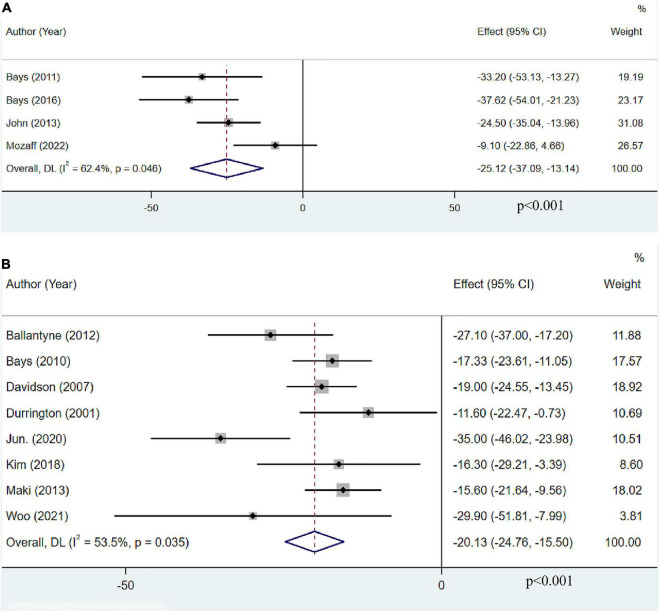
The effect of OM3-FA on VLDL-C. **(A)** OM3-FA monotherapy; **(B)** Combined therapy of statins plus OM3-FA.

Only four articles with 952 participants reported the effect of OM3-FA monotherapy on VLDL-C. The pooled result revealed that OM3-FA significantly decreased VLDL-C levels (MD: −25.12, 95% CI: −37.09 to −13.14; *p* < 0.001), with large heterogeneity (*I*^2^ = 62.4%, *p*_*he*_ = 0.046) (Figure 7A). Eight studies with 1,998 patients assessed the effect of OM3-FA added to statins on VLDL-C. Similar effect was identified (MD: −20.13, 95% CI: −24.76 to −15.50; *p* < 0.001), with obvious heterogeneity (*I*^2^ = 53.5%, *p*_*he*_ = 0.035) (Figure 7B). In subgroup analysis, when the dosage of OM3-FA was <4 g, the pooled result of two studies showed no significant effect on VLDL-C (MD: −23.00, 95% CI: −50.94 to 4.94, *p* = 0.11; *I*^2^ = 85%, *p*_*he*_ = 0.009) (Table 2). For the combined therapy of statins plus OM3-FA, the subgroup analyses were consistent with the overall effect (Table 3).

### The effect of omega-3 fatty acids on non-high-density lipoprotein cholesterol

**FIGURE 8:**
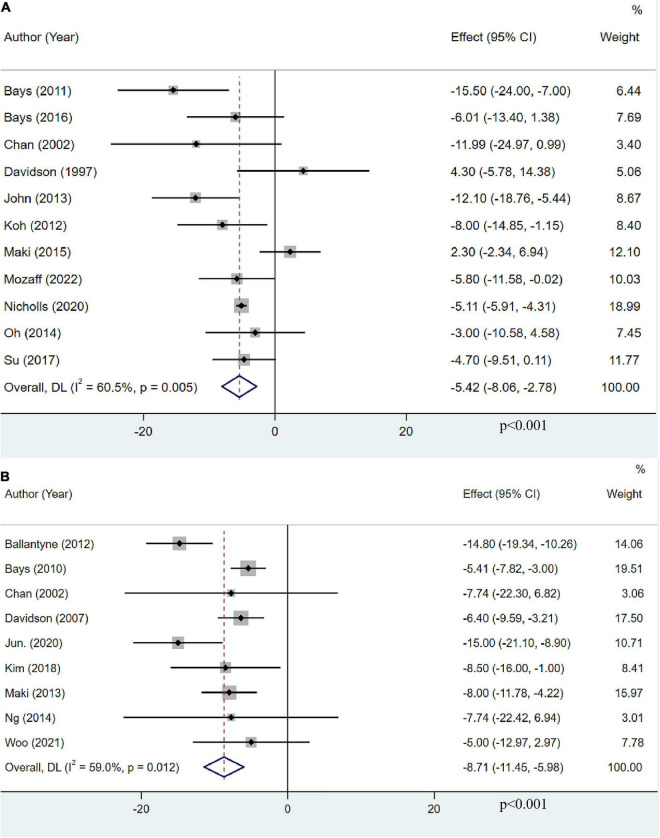
The effect of OM3-FA on non-HDL-C. **(A)** OM3-FA monotherapy; **(B)** Combined therapy of statins plus OM3-FA.

Eleven studies, including 13,179 participants, investigated the effects of OM3-FA on non-HDL-C. The pooled result demonstrated that OM3-FA significantly reduced non-HDL-C level (MD: −5.42, 95% CI: −8.06 to −2.78; *p* < 0.001), with large heterogeneity (*I*^2^ = 60.5%, *p* = 0.005) (Figure 8A). Nine studies with 1,995 patients assessed the effect of OM3-FA added to statins on non-HDL-C. the effect of lowering non-HDL-C was more obvious (MD: −8.71, 95% CI: −11.45 to −5.98; *p* < 0.001), with significant heterogeneity (*I*^2^ = 59%, *p* = 0.012) (Figure 8B). However, subgroup analyses showed that non-HDL-C reduction was statistically significant only when the dose of OM3-FA was ≥4 g (MD:−7.32, 95% CI: −10.67 to −3.96, *p* < 0.0001; *I*^2^ = 55%, *p*_*he*_ = 0.05), EPA and DHA were used together (MD:−5.41, 95% CI: −6.56 to −4.25, *p* < 0.00001; *I*^2^ = 4%, *p*_*he*_ = 0.39), the baseline TG level of participants was ≥500 mg/dL (MD:−9.39, 95% CI: −13.84 to −4.94, *p* < 0.0001; *I*^2^ = 39%, *p*_*he*_ = 0.18) and the duration of treatment was ≥12 weeks (MD: −7.83, 95% CI: −11.48 to −4.18, *p* < 0.0001; *I*^2^ = 59%, *p*_*he*_ = 0.04) (Table 2). When OM3-FA was combined with statins, all subgroup analyses showed statistically significant reductions in non-HDL-C levels except when participants had baseline TG levels of less than 200 mg/dL (MD: −7.74, 95% CI: −18.08 to 2.60, *p* = 0.14; *I*^2^ = 0%, *p*_*he*_ = 1.00) (Table 3).

### The effect of omega-3 fatty acids on apolipoprotein B

**FIGURE 9:**
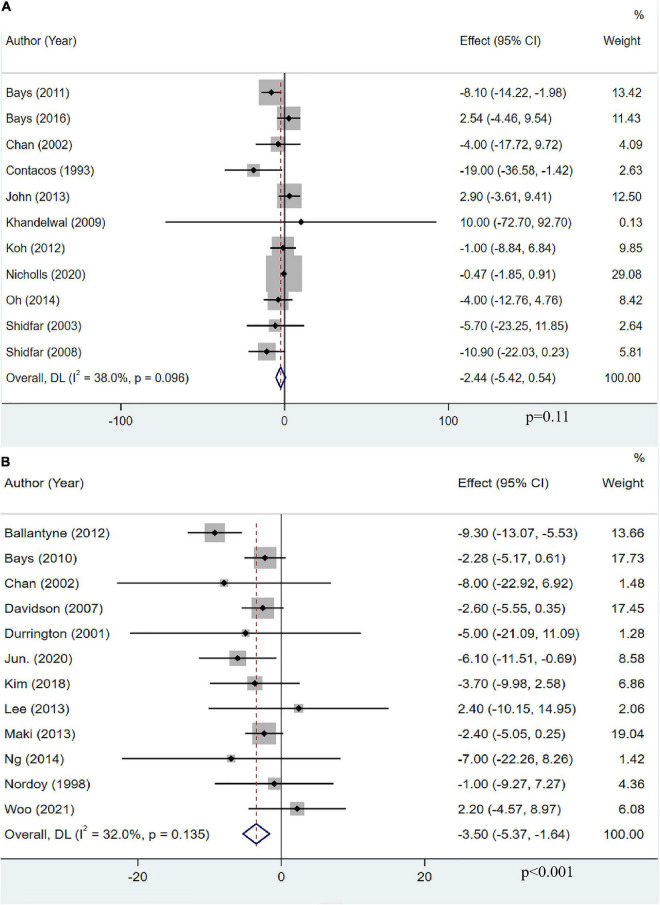
The effect of OM3-FA on Apo-B. **(A)** OM3-FA monotherapy; **(B)** Combined therapy of statins plus OM3-FA.

Eleven studies with 12,563 participants explored the effects of OM3-FA on Apo-B. The pooled result demonstrated that OM3-FA had no significant effect on Apo-B compared with control group (MD: −2.44, 95% CI: −5.42 to 0.54; *p* = 0.11), with low heterogeneity (*I*^2^ = 38%, *p* = 0.096) (Figure 9A). Twelve studies with 2,108 patients assessed the effect of OM3-FA added to statins on Apo-B. However, as opposed to OM3-FA alone, the combination exerted a significant reduction in Apo-B level (MD: −3.50, 95% CI: −5.37 to −1.64; *p* < 0.001) without significant heterogeneity (*I*^2^ = 32%, *p* = 0.135) (Figure 9B). In subgroup analyses, we found that the effect of OM3-FA on reducing Apo-B was significant if the treatment duration was <12 weeks (MD: −5.19, 95% CI: −9.70 to −0.69, *p* = 0.02; *I*^2^ = 0%, *p*_*he*_ = 0.58) (Table 2). When OM3-FA were added to statins, all subgroups showed significant reductions in Apo-B levels except when participants’ baseline TG levels were less than 200 mg/dL (MD: −7.51, 95% CI: −18.18 to 3.16, *p* = 0.17; *I*^2^ = 0%, *p*_*he*_ = 0.93) (Table 3).

### The effect of omega-3 fatty acids on apolipoprotein AI

**FIGURE 10:**
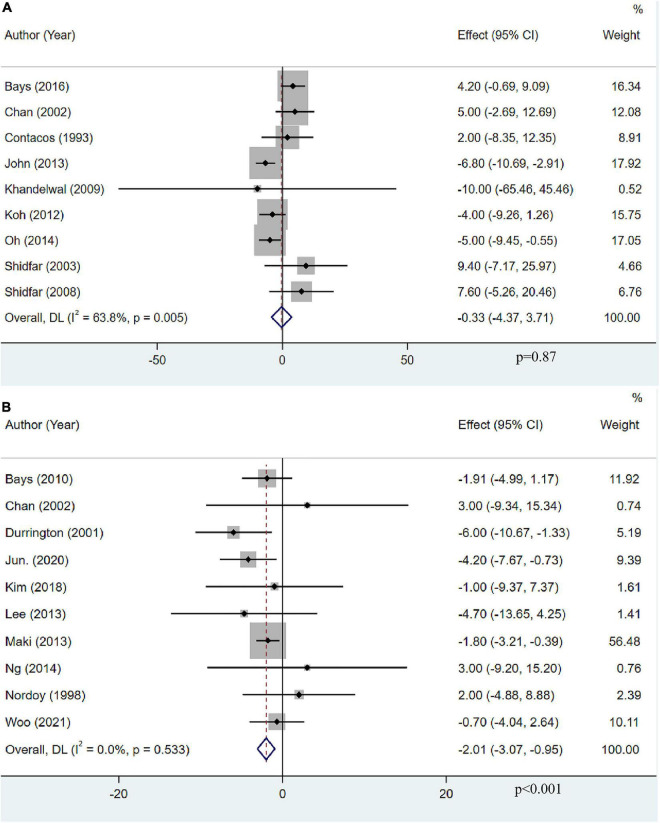
The effect of OM3-FA on Apo-AI. **(A)** OM3-FA monotherapy; **(B)** Combined therapy of statins plus OM3-FA.

Apolipoprotein AI levels were assessed as an outcome measure in nine studies with 684 participants. The pooled result demonstrated that OM3-FA had no significant effect on Apo-AI (MD: −0.33, 95% CI: −4.37 to 3.71; *p* = 0.87), with obvious heterogeneity (*I*^2^ = 63.8%, *p* = 0.005) (Figure 10A). Ten studies with 1,418 patients assessed the effect of OM3-FA added to statins on Apo-AI. Intriguingly, combination therapy exerted a significant reduction in Apo-AI level (MD: −2.01, 95% CI: −3.07 to −0.95; *p* < 0.001), without heterogeneity (*I*^2^ = 0%, *p* = 0.533) (Figure 10B). When OM3-FA was used alone, the results of all subgroup analyses were consistent with the overall result (Table 2). For combination therapy, the results of all subgroup analyses were consistent with the overall results except when participants had baseline TG levels of less than 200 mg/dL (MD: 3.00, 95% CI: −5.68 to 11.68, *p* = 0.50; *I*^2^ = 0%, *p*_*he*_ = 1.00) (Table 3).

### Sensitivity analysis

Sensitivity analyses were performed, in which one study was removed, and the others analyzed to estimate whether the results could have been affected markedly by a single study. The sensitivity analyses’ results indicated no reversals and significant fluctuations in all outcomes except for the effect of OM3-FA monotherapy on HDL-C. After removing one study (11), the sensitivity analysis showed that the 95% CI of the HDL-C narrowed significantly (Supplementary material 3; Figure 3).

### Publication bias

The results of funnel plots and Egger’s tests showed that there might be a publication bias for several outcomes, specifically including the effect of OM3-FA monotherapy on TG (Egger’s test, *p* = 0.071) and LDL-C (Egger’s test, *p* = 0.028) and the impact of combined therapy of statins plus OM3-FA on TG (Egger’s test, *p* = 0.044) and TC (Egger’s test, *p* = 0.049) levels. Consequently, a trim-and-fill method was conducted. Results showed no trimming was performed, and the results were unchanged for the effect on LDL-C with OM3-FA monotherapy and the impact on TG and TC with combined therapy of OM3-FA added to statins. The result was also not reversed for the efficacy of OM3-FA monotherapy on TG after filling in four studies. Therefore, the results of our meta-analysis are all robust. Details were available in Supplementary material 4.

## Discussion

This meta-analysis aimed to evaluate the effects of OM3-FA monotherapy or combined therapy of statins plus OM3-FA on TG and other lipid profiles in patients with hypertriglyceridemia. Through the analysis based on the 32 RCTs, the findings are as follows: first, the efficacy of lowering TG was definite whether OM3-FA monotherapy or combined therapy of statins plus OM3-FA; Second, the levels of TC, VLDL-C, and non-HDL-C also showed a significant reduction both with OM3-FA monotherapy and integrated treatment of OM3-FA and statins. Third, OM3-FA monotherapy elevated LDL-C and HDL-C levels, while combined therapy of statins plus OM3-FA exerted no significant effect on LDL-C and HDL-C. Fourth, the concentrations of Apo-B and Apo-AI were not significantly affected by OM3-FA monotherapy but were significantly reduced by the combined therapy of OM3-FA and statins. Fifth, subgroup analysis demonstrated that DHA significantly increased the level of TC.

Compared to previous studies, a meta-analysis assessing the effect of OM3-FA on type 2 diabetes showed that OM3-FA lowered TG and VLDL-C levels, raised LDL-C levels (51), which was consistent with the results of our research. However, it showed no significant effect on TC, which was different from our results. We speculated that it might be due to the different populations included. In addition, our study showed that OM3-FA at doses ≥4 g or <4 g was effective in reducing triglyceride levels, the same as the results of a meta-analysis investigating the effects of OM3-FA on HIV-associated hypertriglyceridemia (15).

The results found that both OM3-FA monotherapy and combined therapy of OM3-FA with statins reduced TC levels. However, when combined with statins, the reduction in TC was more potent, and we suspected that it was not simply because the statins lowered LDL-C. It is well known that LDL-C, HDL-C, and VLDL-C are all included in TC. This meta-analysis showed that OM3-FA monotherapy increased LDL-C and HDL-C, which did not happen for the combined treatment. Besides, the effect on VLDL-C was similar between two treatment modalities; this undoubtedly increases the gap in total cholesterol reduction. Similarly, both VLDL-C and LDL-C are included in non-HDL-C; therefore, when OM3-FA was added to statins, the reduction in non-HDL-C was also more apparent. However, why does OM3-FA increase LDL-C levels? The following reasons may be explained. Lu. et al. (52) found that OM3-FA could amplify the propensity of very low-density lipoprotein (VLDL) to be converted to low-density lipoprotein (LDL). OM3-FA also diminished hepatic triglyceride-rich lipoprotein (TRL) secretion and enhanced TRL to LDL conversion (53). Moreover, it is reported that DHA enhances VLDL lipolysis, leading to greater conversion to LDL and increasing larger, more buoyant LDL particles (54). Two other studies also suggested that DHA-containing supplements significantly elevated LDL-C (55, 56). In addition, subgroup analysis showed that DHA significantly increased the level of TC, probably also because DHA increased LDL-C levels. Regarding the reason why LDL-C did not increase when OM3-FA was combined with statins, we thought it might be because the LDL-C lowering effect of statins outweighed the impact of OM3-FA in raising LDL-C.

Regarding the effect of OM3-FA on HDL-C, individual studies were found to impact the overall result significantly. After removing one article (11), the heterogeneity of OM3-FA monotherapy on HDL-C was reduced from 56 to 0%. We speculated that this trial could be the source of heterogeneity by combining sensitivity analysis results. However, the outcome of this study was consistent with the pooled results. Therefore, this did not affect the interpretation of the effect of OM3-FA on HDL-C. When OM3-FA was used in combination with statins, the pooled results showed no significant effect on HDL-C; However, when two studies were removed (41, 44), the results were reversed, and heterogeneity was also significantly reduced. Hence, we reviewed these two articles. One study (41) used only EPA, and the other (44) showed significant differences in baseline TG levels between the experimental and control groups (336.7 and 407.6 mg/dL, respectively). Therefore, we speculate that these might be the sources of heterogeneity and thus affect the overall results. The above analyses suggest that OM3-FA are tended to increase HDL-C levels. Nonetheless, the impact of combined therapy of statins plus OM3-FA on HDL-C still needs to be confirmed by large randomized multicenter well-designed trials.

Apolipoprotein B and Apo-AI are the main surface proteins on LDL and HDL particles, respectively (57). The Apo-B/Apo-AI ratio is an essential indicator of atherosclerosis. Our results showed that OM3-FA monotherapy had no significant effect on Apo-B and Apo-AI. However, when combined with statins, the levels of Apo-B and Apo-AI were significantly reduced compared to the control group. It was reported that statins affect lipid metabolism primarily by inhibiting cholesterol biosynthesis, which in turn increases the number of hepatic Apo-B receptors, and the clearance of all Apo B-containing LDL and VLDL is accelerated when OM3-FA is co-administrated (58). However, why the level of Apo-AI decreased after OM3-FA was added to statins needs to be further explored. Since combination therapy with OM3-FA and statins reduced Apo-B and Apo-AI simultaneously, the ratio may not change much. Therefore, we thought that combination therapy for the treatment of hypertriglyceridemia is still desirable.

We thought some implications from this meta-analysis could be obtained to guide clinical practice. First, when OM3-FA is used alone, if the patient’s LDL-C is already at a high level, it is not recommended because of the risk of further increasing LDL-C. Second, OM3-FA can be added to statins to treat residual hypertriglyceridemia, thus lowering TG without increasing LDL-C levels. Third, because of the risk of raising total cholesterol, DHA should be used in combination with EPA.

This meta-analysis has several strengths. First, compared with the previous meta-analysis, we included more literature with a larger sample size, making our results more robust. Second, nearly half of the included articles were multicenter, randomized, double-blind experiments, which made our results more accurate. Third, our study included populations from all over the world, which increased the generalizability of the results. Fourth, most of the included studies provided dietary guidance and advice to the participants, which significantly reduced the influence of dietary habits on the findings. Fifth, the studies we included all had a parallel design, which significantly reduced the influence of the study method on the results. Lastly, our study not only investigated the effect of OM3-FA on different blood lipid profiles but also evaluated the effect of the combination of OM3-FA and statins on lipid profiles, to some extent, which provided the necessary guidance for the correct use of OM3-FA.

However, several limitations of our study cannot be overlooked. First, significant heterogeneity was identified in most of the results even though we performed subgroup analyses according to intervention time, baseline TG level of participants, and type and dosage of OM3-FA. Fortunately, sensitivity analysis found that individual studies did not affect most outcomes. Second, the statins used varied across articles. Some studies did not indicate the type of statins and only generally referred to statins treatment, which greatly limited the subgroup analysis based on the type of statin. Third, we failed to investigate further the dose-response relationship of the effects of OM3-FA on lipid profiles.

Based on the current study, there are some recommendations for future studies on OM3-FA. The dose-response relationship of OM3-FA needs to be further investigated to provide a suitable and effective starting dose. The adverse effects of OM3-FA compared with placebo also require further evaluation. This study only evaluated the impact of OM3-FA on lipid profile, and whether there are some advantages of OM3-FA compared with other triglyceride-lowering drugs also needs to be further explored to provide an appropriate option for patients with hypertriglyceridemia.

## Conclusion

In conclusion, the results of our meta-analysis indicated that OM3-FA monotherapy could decrease the concentrations of TG, TC, VLDL-C, and non-HDL-C and increase the levels of LDL-C and HDL-C, without significant effects on Apo-B and Apo-AI. The combined therapy of statins plus OM3-FA could exert significant reductions in TG, TC, VLDL-C, non-HDL-C, Apo-B, and Apo-AI levels, with no significant impact on LDL-C and HDL-C. Nevertheless, the effects of OM3-FA observed in this review should be interpreted with caution due to the high heterogeneity between the included studies.

## Data availability statement

The original contributions presented in this study are included in the article/Supplementary material, further inquiries can be directed to the corresponding author/s.

## Author contributions

YY and WD conceived the study, designed the search strategy, conducted the study selection, interpreted the results, and drafted the manuscript. YW and TL extracted the data and performed the statistical analyses. YC and CL evaluated the risk of bias of included studies. QW and YW processed the pictures and tables. QC provided the guidance and resolved disagreements. All authors read and approved the final version of the manuscript.

## Footnotes

1
https://www.crd.york.ac.uk/prospero/

2
ClinicalTrials.gov

3
www.training.cochrane.org/handbook
