# Supplementary material for: The effect of omega-3 fatty acids and its combination with statins on lipid profile in patients with hypertriglyceridemia: A systematic review and meta-analysis of randomized controlled trials
Source: Front Nutr. 2022 Oct 13;9:1039056. doi: 10.3389/fnut.2022.1039056 (PMC9609787; doi:10.3389/fnut.2022.1039056)
Supplement: Supplementary file 1 [file Data_Sheet_1.zip › Supplementary materials 2 Search strategies.DOCX]

**Pubmed**

((("Hypertriglyceridemia"[Mesh]) OR (((((((" Hyperlipoproteinemia Type IV ") OR ("Type V Hypertriglyceridemic Waist")) OR (Hyperlipidemia)) OR (hyperlipemia)) OR (HLP)) OR ("high fat blood disease")) OR ("hypertriglyceridemisome sort of "))) AND (("Fatty Acids, Omega-3"[Mesh]) OR ((((((((((((((((((((((((((((((((Omega-3 Fatty Acid) OR (Acid, Omega-3 Fatty)) OR (Fatty Acid, Omega-3)) OR (Omega 3 Fatty Acid)) OR (Omega-3 Fatty Acids)) OR (n-3 Oil)) OR (Oil, n-3)) OR (n 3 Oil)) OR (Oil, n3)) OR (n-3 Fatty Acids)) OR (n 3 Fatty Acids)) OR (Omega 3 Fatty Acids)) OR (n-3 PUFA)) OR (PUFA, n-3)) OR (n 3 PUFA)) OR (n3 Fatty Acid)) OR (Fatty Acid, n3)) OR (n3 PUFA)) OR (PUFA, n3)) OR (n3 Polyunsaturated Fatty Acid)) OR (n3 Oils)) OR (n-3 Oils)) OR (n 3 Oils)) OR (N-3 Fatty Acid)) OR (Acid, N-3 Fatty)) OR (Fatty Acid, N-3)) OR (N 3 Fatty Acid)) OR (n-3 Polyunsaturated Fatty Acid)) OR (n 3 Polyunsaturated Fatty Acid)) OR (alpha-Linolenic Acid)) OR (Docosahexaenoic Acids)) OR (Eicosapentaenoic Acid)))) AND (((((randomized controlled trial) OR (RCT)) OR (randomized clinical trial)) OR (randomized)) OR (randomly)) 541

**Cochrane library**

ID Search

#1 MeSH descriptor: [Hypertriglyceridemia] explode all trees

#2 (Hyperlipidemia):ti,ab,kw OR (hyperlipemia):ti,ab,kw OR (HLP):ti,ab,kw OR (high fat blood disease):ti,ab,kw (Word variations have been searched)

#3 #1 OR #2

#4 MeSH descriptor: [Fatty Acids, Omega-3] explode all trees

#5 (Omega-3 Fatty Acid):ti,ab,kw OR (Acid, Omega-3 Fatty):ti,ab,kw OR (Fatty Acid, Omega-3):ti,ab,kw OR (Omega 3 Fatty Acid):ti,ab,kw OR (Omega-3 Fatty Acids):ti,ab,kw (Word variations have been searched)

#6 (n-3 Oil):ti,ab,kw OR (Oil, n-3):ti,ab,kw OR (n 3 Oil):ti,ab,kw OR (Oil, n3):ti,ab,kw OR (n-3 Fatty Acids):ti,ab,kw (Word variations have been searched)

#7 (n 3 Fatty Acids):ti,ab,kw OR (Omega 3 Fatty Acids):ti,ab,kw OR (n-3 PUFA):ti,ab,kw OR (PUFA, n-3):ti,ab,kw OR (n 3 PUFA):ti,ab,kw (Word variations have been searched)

#8 (n3 Fatty Acid):ti,ab,kw OR (Fatty Acid, n3):ti,ab,kw OR (n3 PUFA):ti,ab,kw OR (PUFA, n3):ti,ab,kw OR (n3 Polyunsaturated Fatty Acid):ti,ab,kw (Word variations have been searched)

#9 (n3 Oils):ti,ab,kw OR (n-3 Oils):ti,ab,kw OR (n 3 Oils):ti,ab,kw OR (N-3 Fatty Acid):ti,ab,kw OR (Acid, N-3 Fatty):ti,ab,kw (Word variations have been searched)

#10 (Fatty Acid, N-3):ti,ab,kw OR (N 3 Fatty Acid):ti,ab,kw OR (n-3 Polyunsaturated Fatty Acid):ti,ab,kw OR (n 3 Polyunsaturated Fatty Acid):ti,ab,kw OR (alpha-Linolenic Acid):ti,ab,kw (Word variations have been searched)

#11 (Docosahexaenoic Acids):ti,ab,kw OR (Eicosapentaenoic Acid):ti,ab,kw (Word variations have been searched)

#12 #4 OR #5 OR #6 OR #7 OR #8 OR #9 OR #10 OR #11

#13 (randomized controlled trial):ti,ab,kw OR (RCT):ti,ab,kw OR (randomized clinical trial):ti,ab,kw OR (randomized):ti,ab,kw OR (randomly):ti,ab,kw (Word variations have been searched)

#14 #3 AND #12 AND #13 588

**Embase**

#1 'omega-3 fatty acid'/exp OR 'omega-3 fatty acid' OR (('omega 3'/exp OR 'omega 3') AND fatty AND ('acid'/exp OR acid)) OR (acid, AND 'omega 3' AND fatty) OR (fatty AND acid, AND 'omega 3') OR (omega AND 3 AND fatty AND acid) OR ('omega 3' AND fatty AND acids) OR ('n 3' AND oil) OR (oil, AND 'n 3') OR (oil, AND n3) OR ('n 3' AND fatty AND acids) OR (n AND 3 AND fatty AND acids) OR (omega AND 3 AND fatty AND acids) OR ('n 3' AND pufa) OR (pufa, AND 'n 3') OR (n AND 3 AND pufa) OR (n3 AND fatty AND acid) OR (n3 AND pufa) OR (pufa, AND n3) OR (n3 AND polyunsaturated AND fatty AND acid) OR (n3 AND oils) OR ('n 3' AND oils) OR (n AND 3 AND oils) OR ('n 3' AND fatty AND acid) OR (acid, AND 'n 3' AND fatty) OR (n AND 3 AND fatty AND acid) OR ('n 3' AND polyunsaturated AND fatty AND acid) OR ('alpha linolenic' AND acid) OR (docosahexaenoic AND acids) OR (eicosapentaenoic AND acid) 180557

#2 'hypertriglyceridemia'/exp OR hypertriglyceridemia OR hyperlipidemia OR hyperlipemia OR hlp OR (high AND fat AND blood AND disease) OR (hypertriglyceridemisome AND sort AND of) 155846

#3 'randomized controlled trial'/exp OR 'randomized controlled trial' OR (randomized AND controlled AND ('trial'/exp OR trial)) OR rct OR (randomized AND clinical AND trial) OR randomized OR randomly 1617388

#4 #1 AND #2 AND #3 1403

**Wib of sicence**

#1 ((((((((((((((((((((((((((((((((TS=(Fatty Acids, Omega-3)) OR TS=(Omega-3 Fatty Acid)) OR TS=(Acid, Omega-3 Fatty)) OR TS=(Fatty Acid, Omega-3)) OR TS=(Omega 3 Fatty Acid)) OR TS=(Omega-3 Fatty Acids)) OR TS=(n-3 Oil)) OR TS=(Oil, n-3)) OR TS=(n 3 Oil)) OR TS=(Oil, n3)) OR TS=(n-3 Fatty Acids)) OR TS=(n 3 Fatty Acids)) OR TS=(Omega 3 Fatty Acids)) OR TS=(n-3 PUFA)) OR TS=(PUFA, n-3)) OR TS=(n 3 PUFA)) OR TS=(n3 Fatty Acid)) OR TS=(Fatty Acid, n3)) OR TS=(n3 PUFA)) OR TS=(PUFA, n3)) OR TS=(n3 Polyunsaturated Fatty Acid)) OR TS=(n3 Oils)) OR TS=(n-3 Oils)) OR TS=(n 3 Oils)) OR TS=(N-3 Fatty Acid)) OR TS=(Acid, N-3 Fatty)) OR TS=(Fatty Acid, N-3)) OR TS=(N 3 Fatty Acid)) OR TS=(n-3 Polyunsaturated Fatty Acid)) OR TS=(n 3 Polyunsaturated Fatty Acid)) OR TS=(alpha-Linolenic Acid)) OR TS=(Docosahexaenoic Acids)) OR TS=(Eicosapentaenoic Acid) 57166

#2 ((((TS=(Hypertriglyceridemia)) OR TS=(Hyperlipidemia)) OR TS=(hyperlipemia)) OR TS=(HLP)) OR TS=(high fat blood disease) 28705

#3 (((ALL=(randomized controlled trial)) OR ALL=(randomized controlled trial)) OR ALL=(randomized)) OR ALL=(randomly) 730856

#4 #1 AND #2 AND #3 318
