# Supplementary material for: The effect of omega-3 fatty acids and its combination with statins on lipid profile in patients with hypertriglyceridemia: A systematic review and meta-analysis of randomized controlled trials
Source: Front Nutr. 2022 Oct 13;9:1039056. doi: 10.3389/fnut.2022.1039056 (PMC9609787; doi:10.3389/fnut.2022.1039056)
Supplement: Supplementary file 1 [file Data_Sheet_1.zip › Supplementary materials 3 Sensitivity analysis.docx]

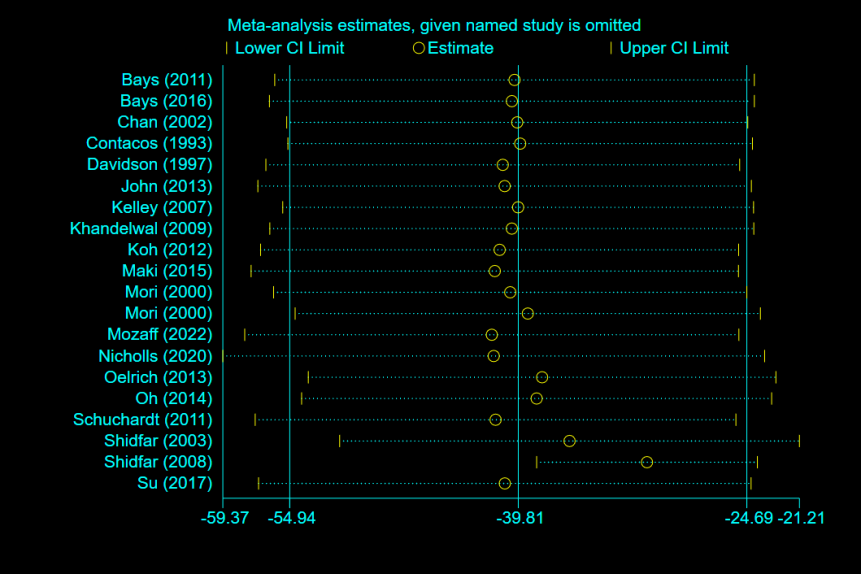


**Figure 1.** Sensitivity analysis of the effect of OM3-FA monotherapy on **TG**


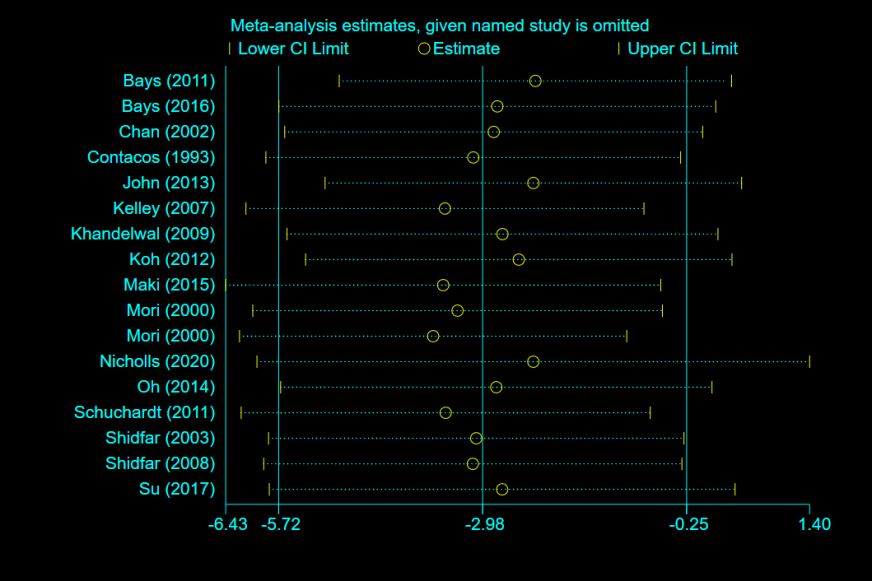


**Figure 2.** Sensitivity analysis of the effect of OM3-FA monotherapy on **TC**


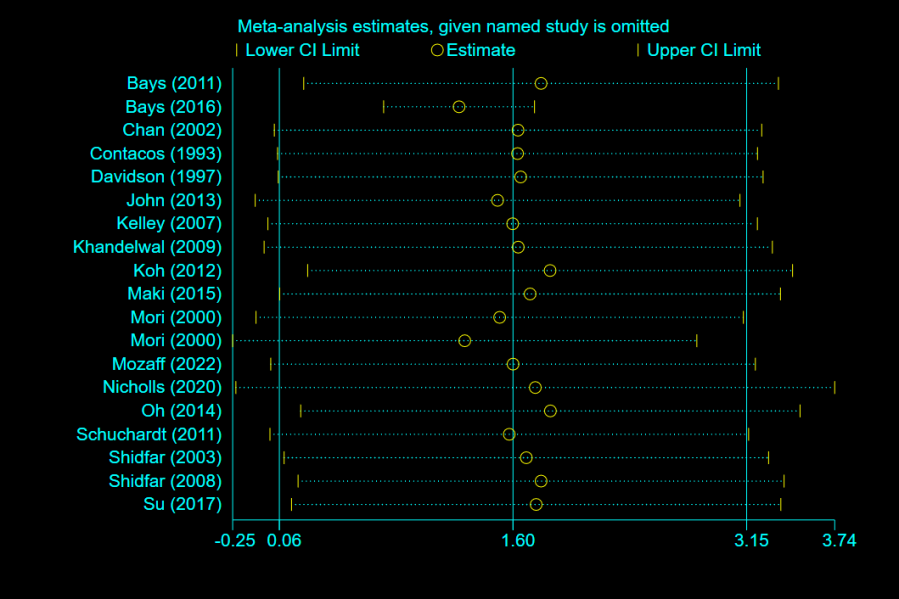


**Figure 3.** Sensitivity analysis of the effect of OM3-FA monotherapy on **HDL-C**
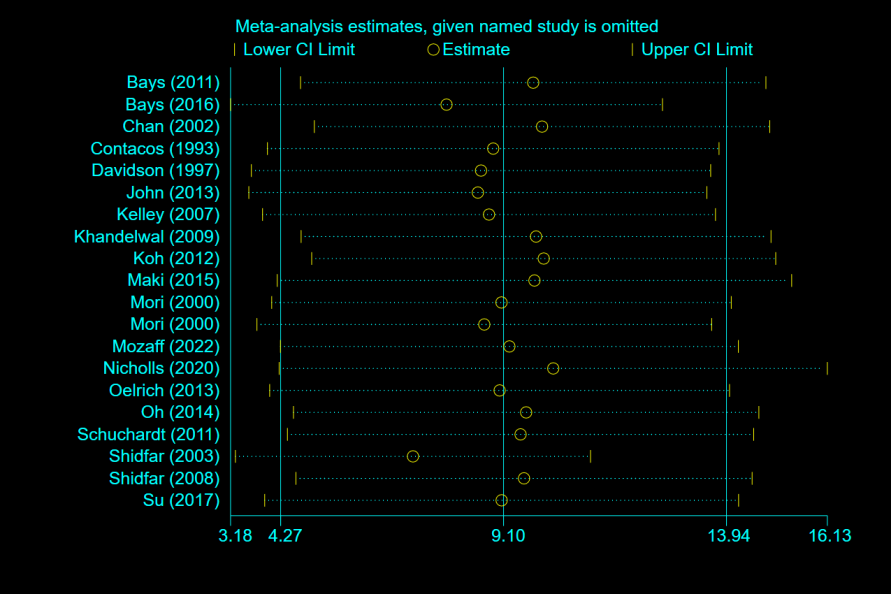


**Figure 4.** Sensitivity analysis of the effect of OM3-FA monotherapy on **LDL-C**


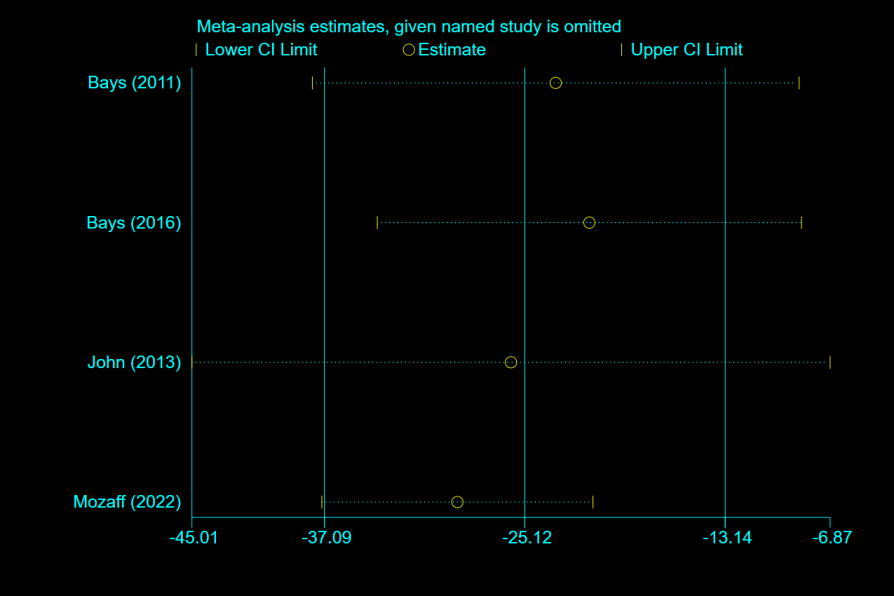


**Figure 5.** Sensitivity analysis of the effect of OM3-FA monotherapy on **VLDL-C**


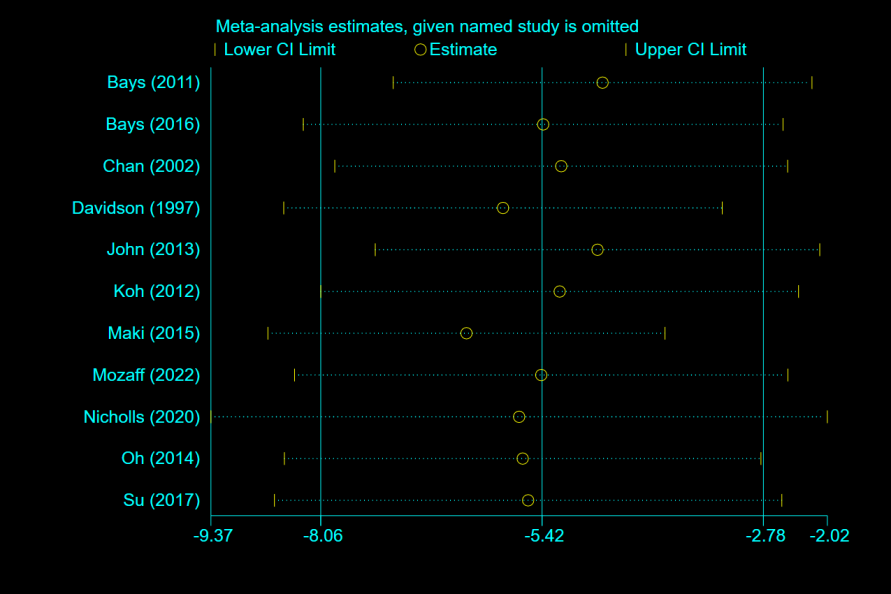


**Figure 6.** Sensitivity analysis of the effect of OM3-FA monotherapy on **non-HDL-C**


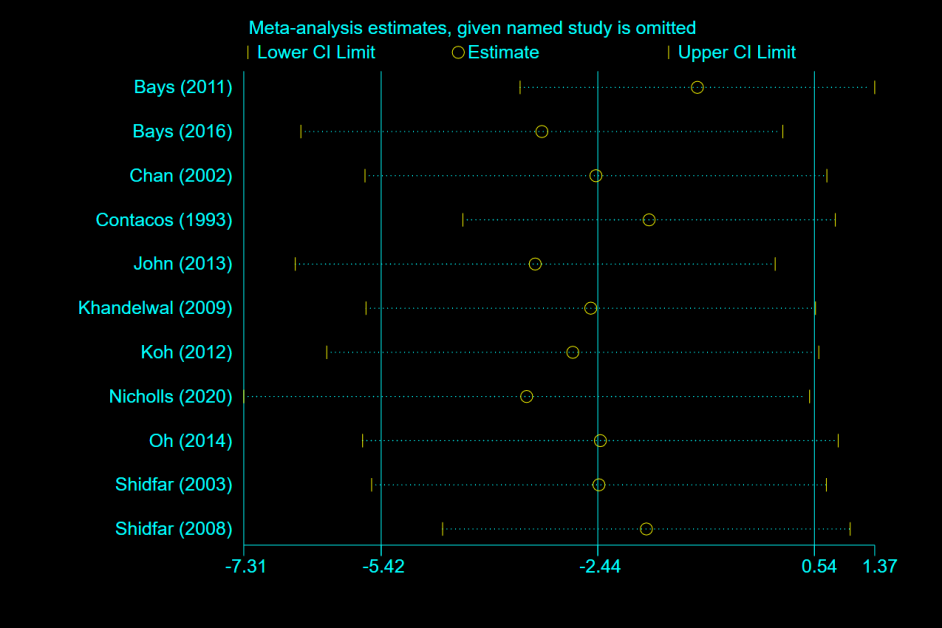


**Figure 7.** Sensitivity analysis of the effect of OM3-FA monotherapy on **Apo-B**


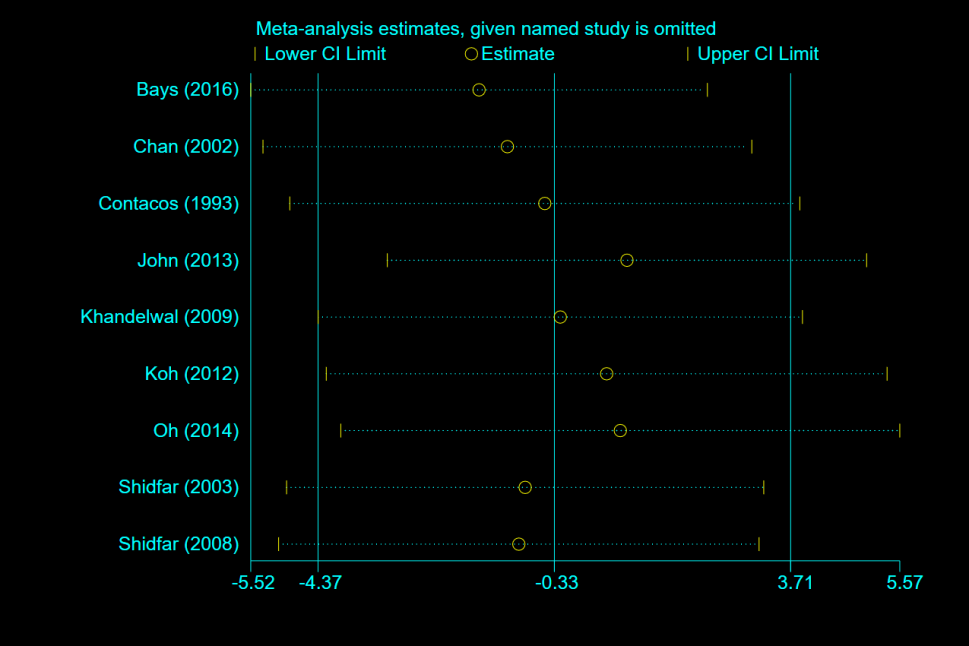


**Figure 8.** Sensitivity analysis of the effect of OM3-FA monotherapy on **Apo-A1**


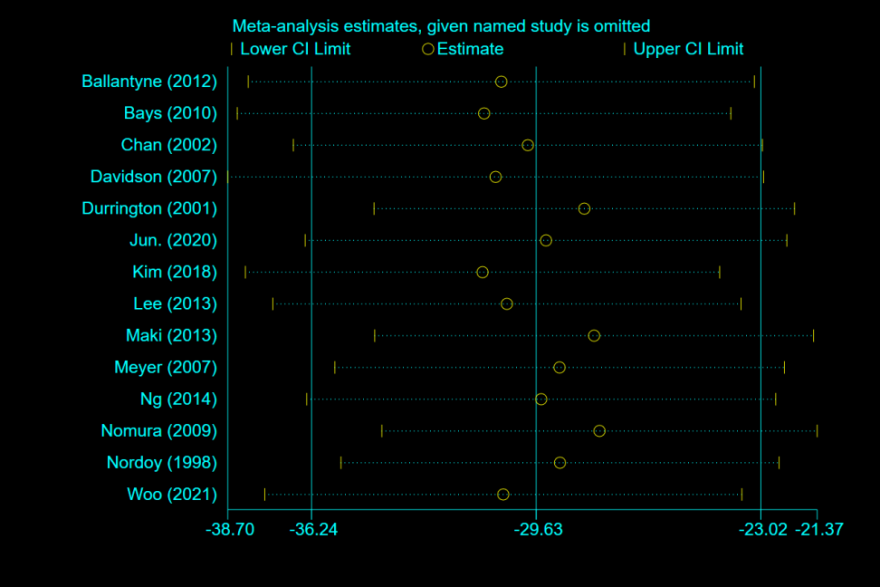


**Figure 9.** Sensitivity analysis of the effect of combined therapy of OM3-FA and statins on **TG**


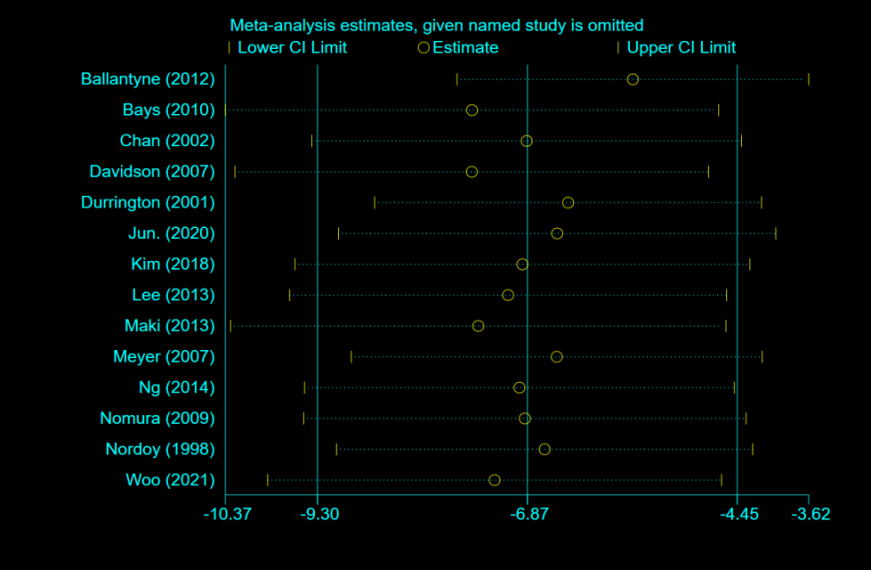


**Figure 10.** Sensitivity analysis of the effect of combined therapy of OM3-FA and statins on **TC**


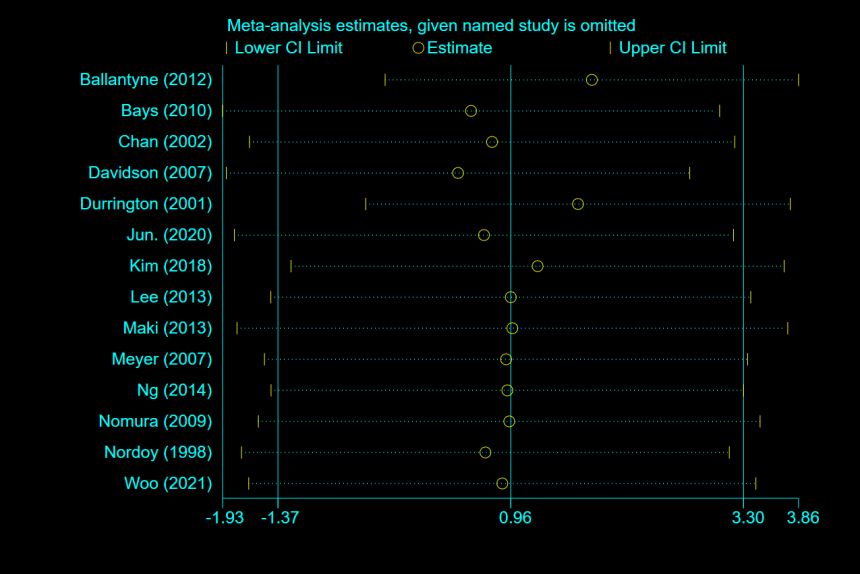


**Figure 11.** Sensitivity analysis of the effect of combined therapy of OM3-FA and statins on **HDL-C**


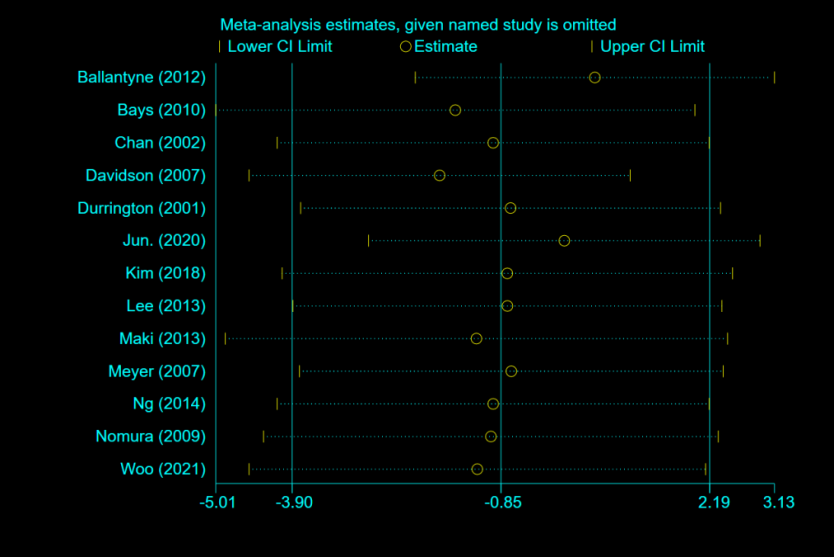


**Figure 12.** Sensitivity analysis of the effect of combined therapy of OM3-FA and statins on **LDL-C**


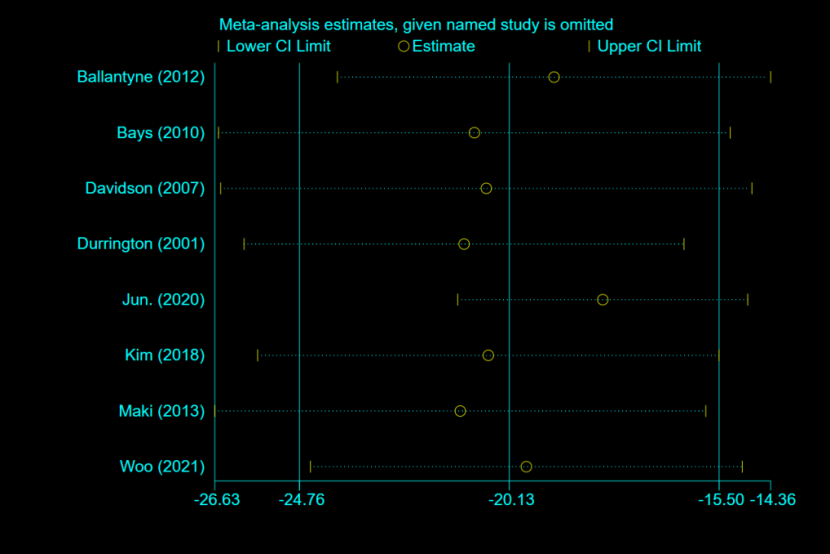


**Figure 13.** Sensitivity analysis of the effect of combined therapy of OM3-FA and statins on **VLDL-C**

**
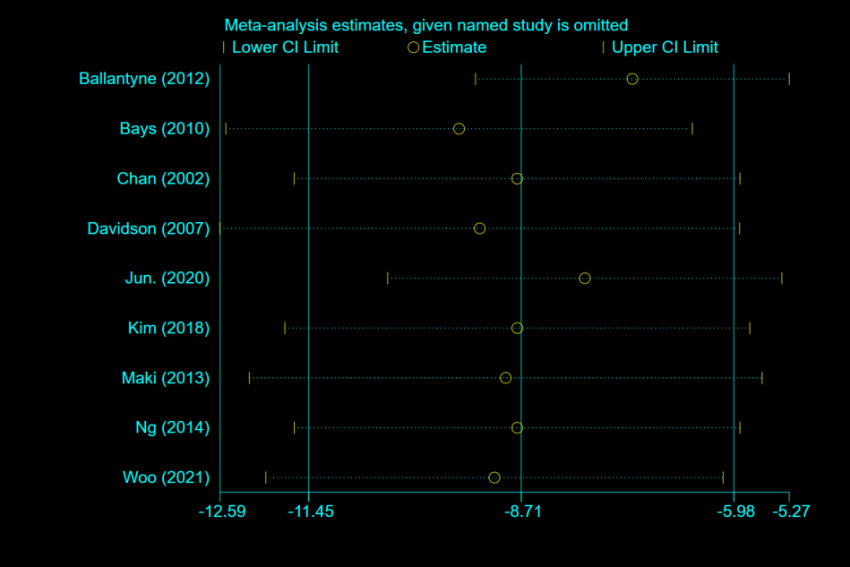
**

**Figure 14.** Sensitivity analysis of the effect of combined therapy of OM3-FA and statins on **non-HDL-C**

**
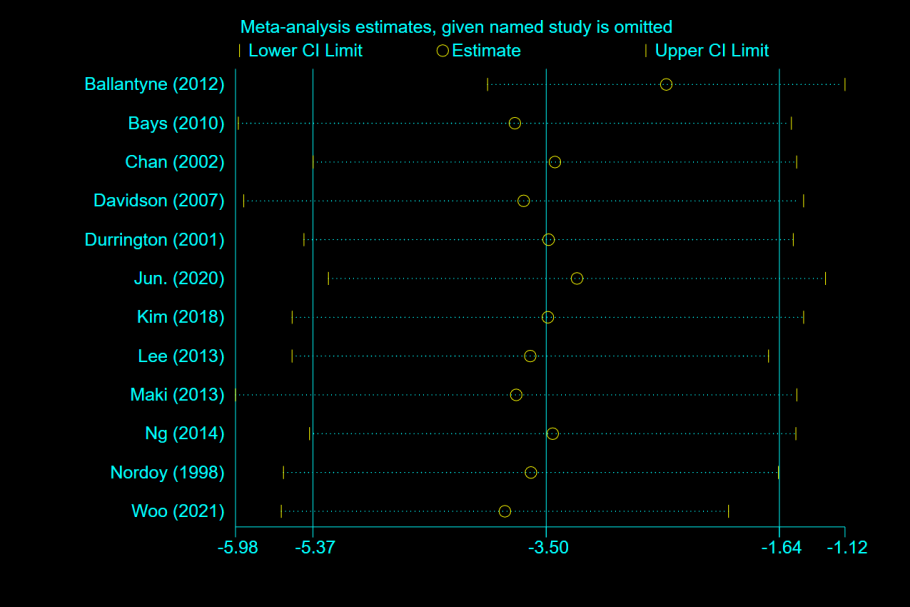
**

**Figure 15.** Sensitivity analysis of the effect of combined therapy of OM3-FA and statins on **Apo-B**


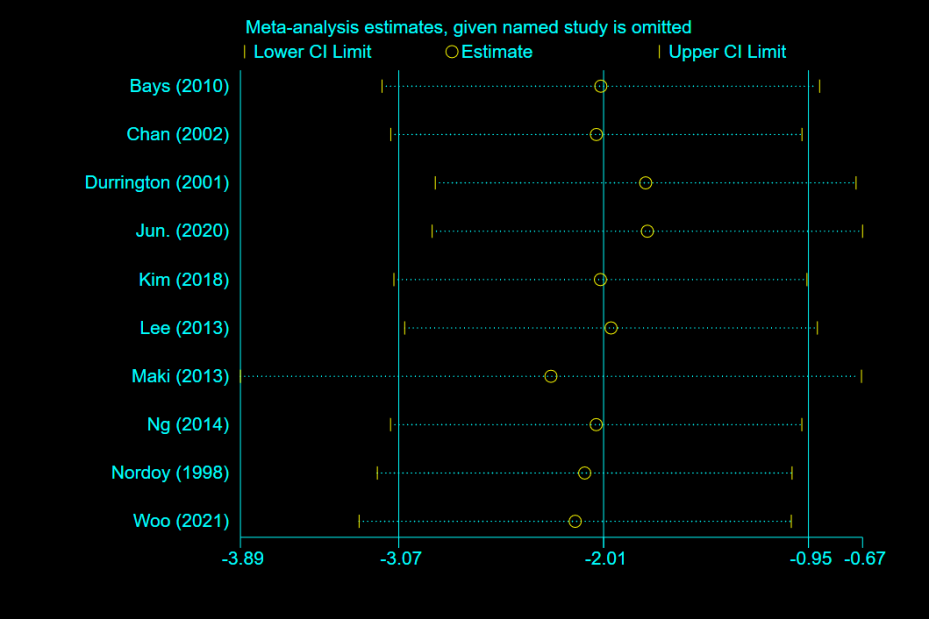


**Figure 16.** Sensitivity analysis of the effect of combined therapy of OM3-FA and statins on **Apo-A1**
