# Supplementary material for: The effect of omega-3 fatty acids and its combination with statins on lipid profile in patients with hypertriglyceridemia: A systematic review and meta-analysis of randomized controlled trials
Source: Front Nutr. 2022 Oct 13;9:1039056. doi: 10.3389/fnut.2022.1039056 (PMC9609787; doi:10.3389/fnut.2022.1039056)
Supplement: Supplementary file 1 [file Data_Sheet_1.zip › Supplementary materials 4 Publication bias.DOCX]

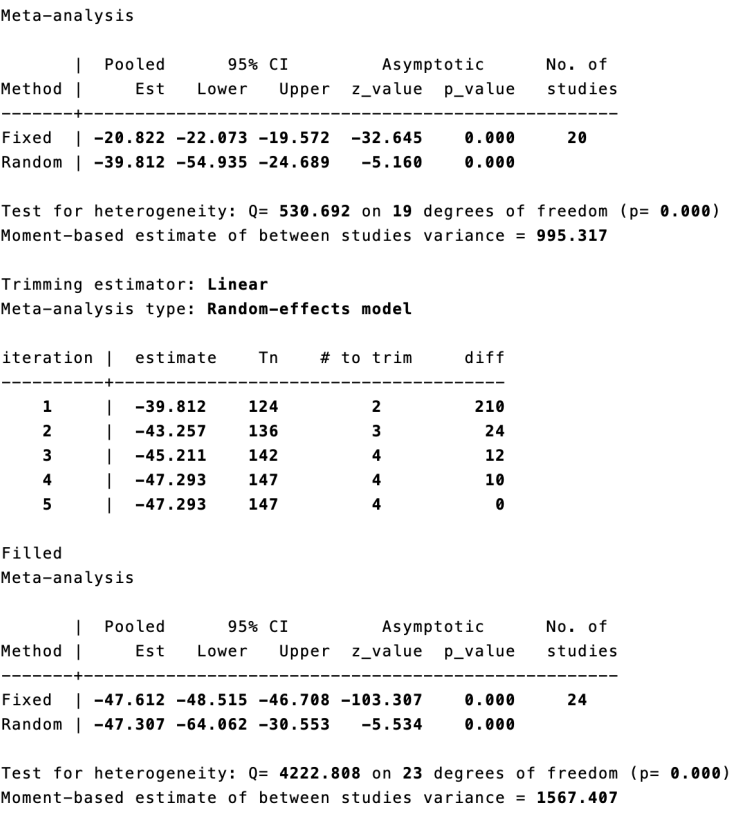

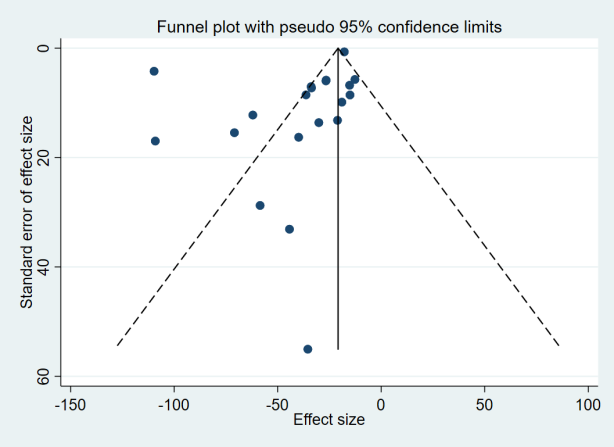

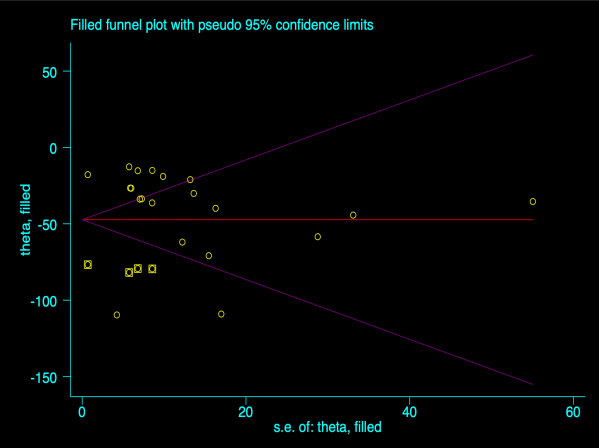


a

b

c

**Figure 1.**  (a) Funnel plot for the effect of OM3-FA monotherapy TG (Egger^,^s test, p=0.071) ; (b) Funnel plot after trimming and filling; (c)the result after trimming and filling


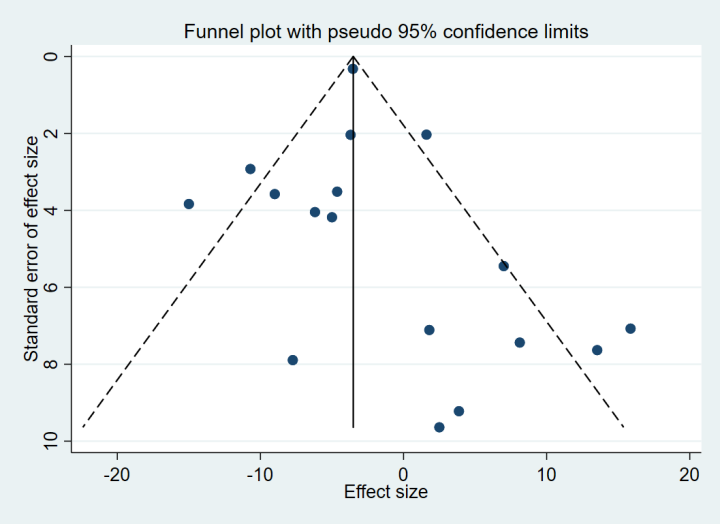


**Figure 2.** Funnel plot for the effect of OM3-FA monotherapy on **TC** (Egger^,^s test, p=0.506)


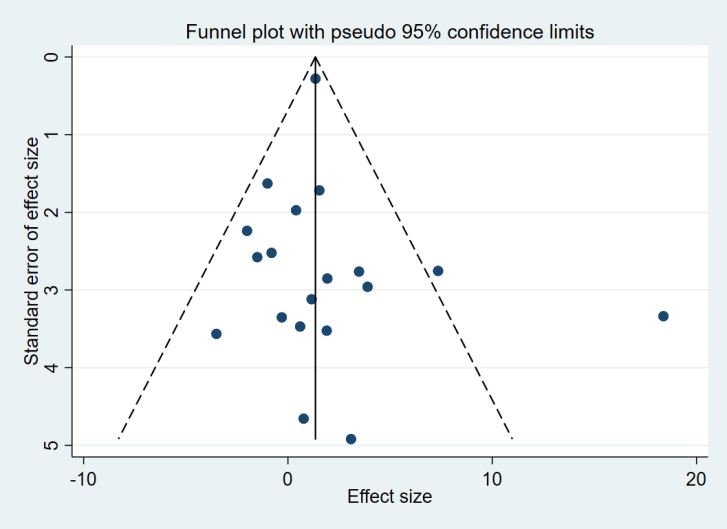


**Figure 3.** Funnel plot for the effect of OM3-FA monotherapy on **HDL-C** (Egger^,^s test, p=0.698)


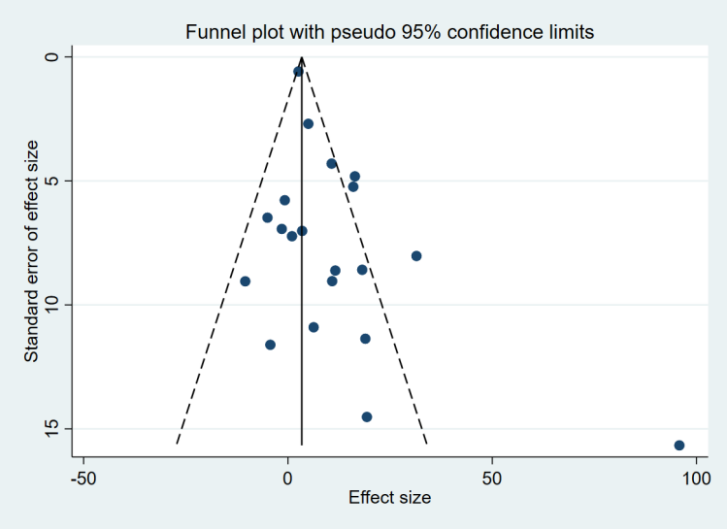


**Figure 4.** Funnel plot for the effect of OM3-FA monotherapy on **LDL-C** (Egger^,^s test, p=0.028)


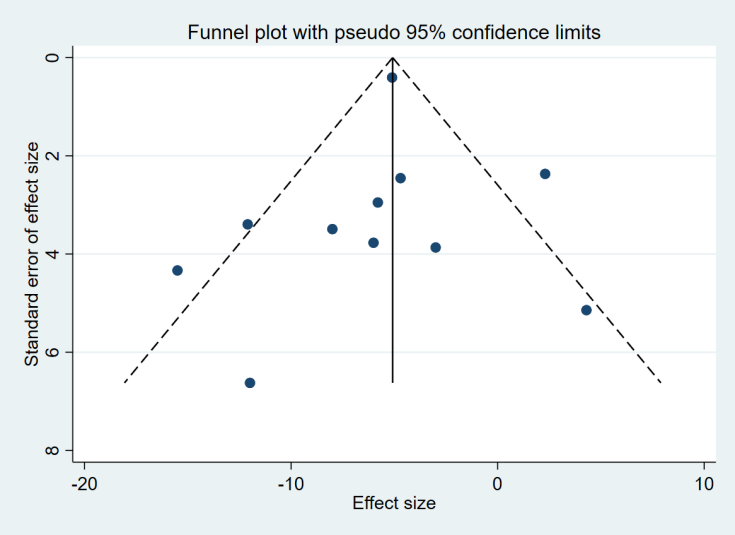


**Figure 5.** Funnel plot for the effect of OM3-FA monotherapy on **non-HDL-C** (Egger^,^s test, p=0.779)


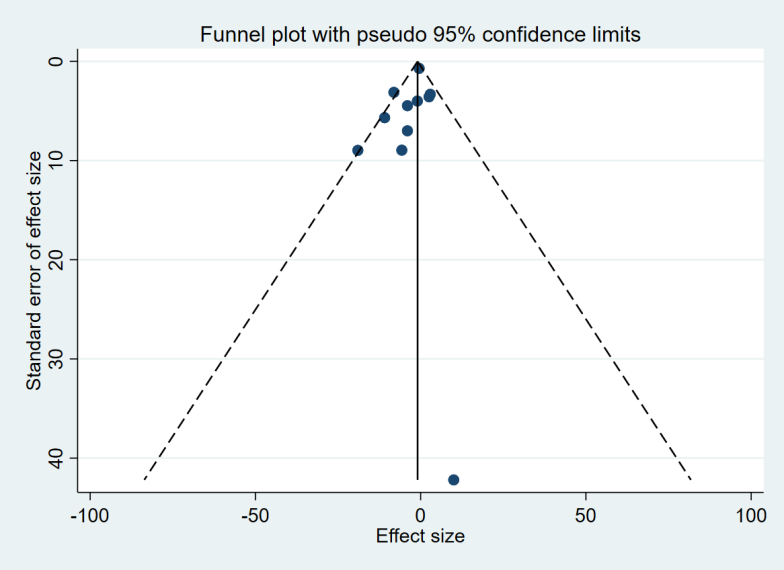


**Figure 6.** Funnel plot for the effect of OM3-FA monotherapy on **Apo-B** (Egger^,^s test, p=0.152)


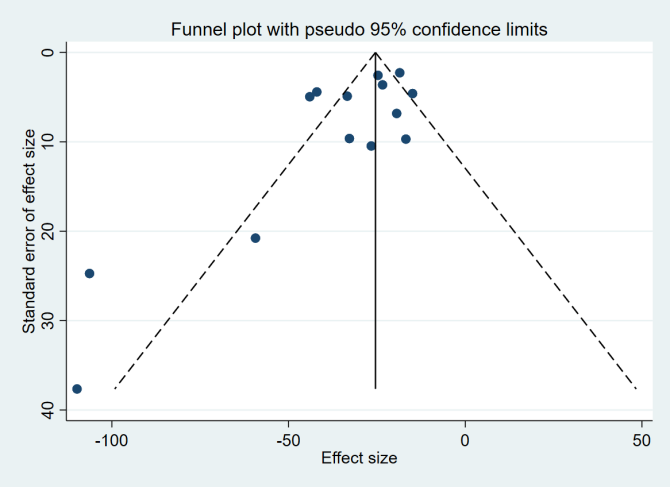


**Figure7.** Funnel plot for the effect of combined therapy of OM3-FA and statins on **TG** (Egger^,^s test, p=0.044)


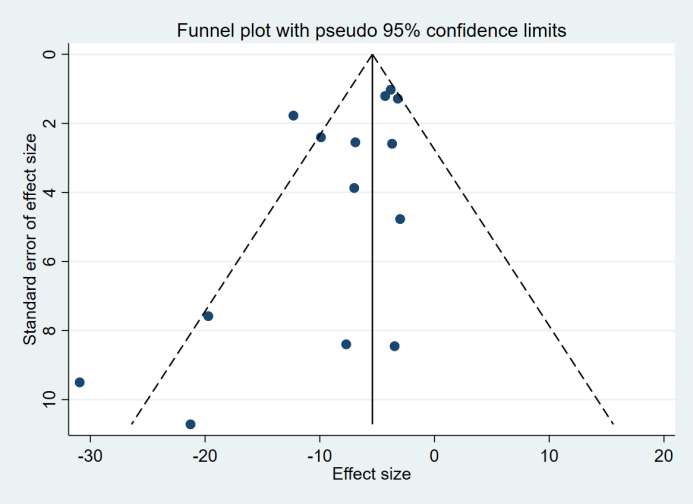


**Figure 8.** Funnel plot for the effect of combined therapy of OM3-FA and statins on **TC** (Egger^,^s test, p=0.049)


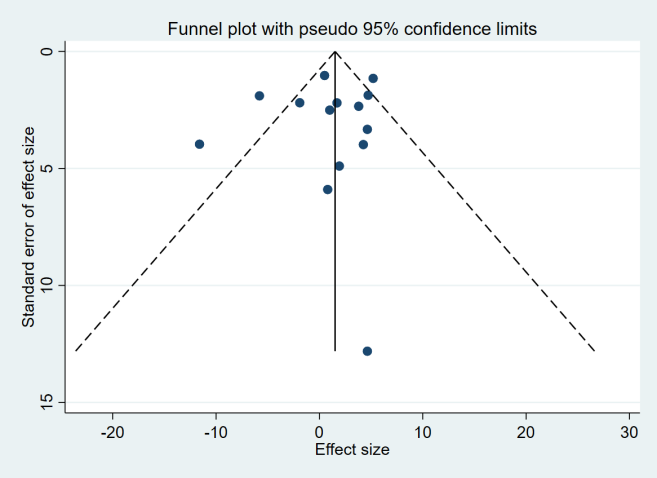


**Figure 9****.** Funnel plot for the effect of combined therapy of OM3-FA and statins on **HDL-C** (Egger^,^s test, p=0.565)


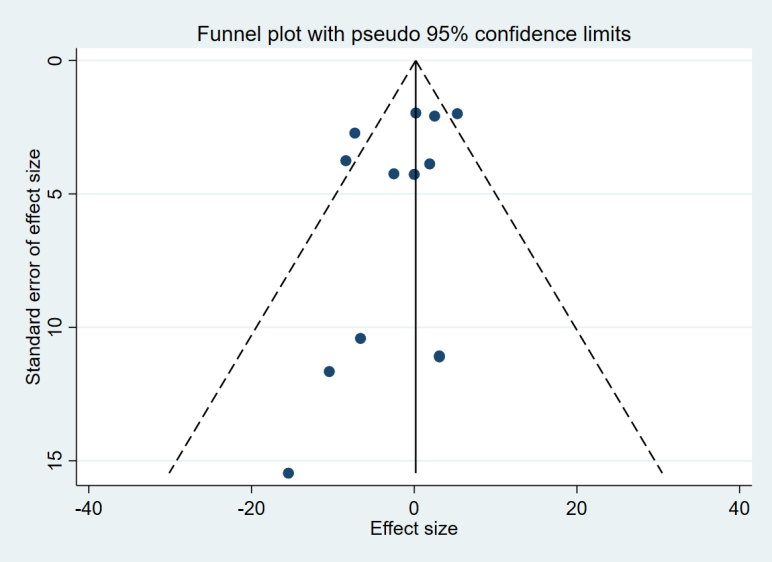


**Figure 10.** Funnel plot for the effect of combined therapy of OM3-FA and statins on **LDL-C** (Egger^,^s test, p=0.191)

**
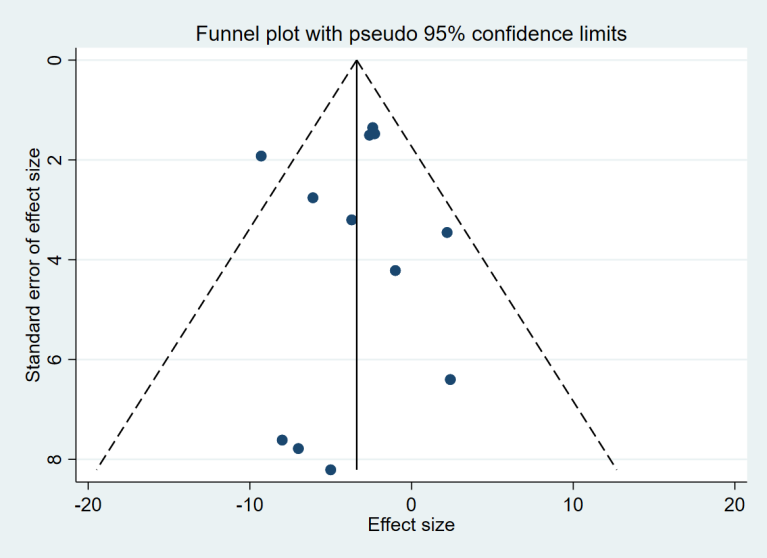
**

**Figure 11.** Funnel plot for the effect of combined therapy of OM3-FA and statins on **Apo-B** (Egger^,^s test,p=0.895)


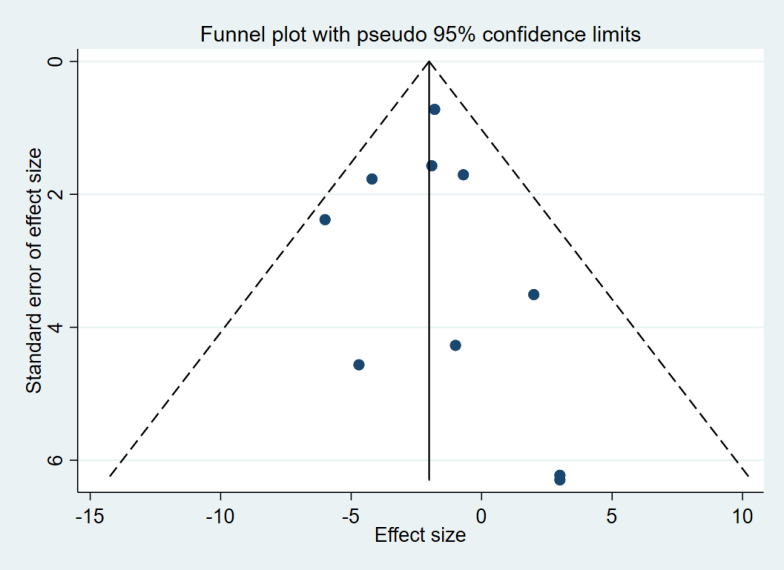


**Figure 12.** Funnel plot for the effect of combined therapy of OM3-FA and statins on **Apo-A1** (Egger^,^s test, p=0.759)
